# Identification of the Wnt signal peptide that directs secretion on extracellular vesicles

**DOI:** 10.1126/sciadv.ado5914

**Published:** 2024-12-11

**Authors:** Uxia Gurriaran-Rodriguez, David Datzkiw, Leandro G. Radusky, Marie Esper, Ehsan Javandoost, Fan Xiao, Hong Ming, Solomon Fisher, Alberto Marina, Yves De Repentigny, Rashmi Kothary, Mikel Azkargorta, Felix Elortza, Adriana L. Rojas, Luis Serrano, Aitor Hierro, Michael A. Rudnicki

**Affiliations:** ^1^ Ottawa Hospital Research Institute, Regenerative Medicine Program, Ottawa, Ontario, Canada.; ^2^ Department of Cellular and Molecular Medicine, Faculty of Medicine, University of Ottawa, Ottawa, Ontario, Canada.; ^3^ Centre for Genomic Regulation (CRG), The Barcelona Institute for Science and Technology, Barcelona, Spain.; ^4^ Center for Cooperative Research in Biosciences (CIC bioGUNE), Basque Research and Technology Alliance (BRTA), Derio, Spain.; ^5^ Institució Catalana de Recerca i Estudis Avançats (ICREA), Barcelona, Spain.; ^6^ IKERBASQUE, Basque Foundation for Science, Bilbao, Spain.

## Abstract

Wnt proteins are hydrophobic glycoproteins that are nevertheless capable of long-range signaling. We found that Wnt7a is secreted long distance on the surface of extracellular vesicles (EVs) following muscle injury. We defined a signal peptide region in Wnts required for secretion on EVs, termed exosome-binding peptide (EBP). Addition of EBP to an unrelated protein directed secretion on EVs. Palmitoylation and the signal peptide were not required for Wnt7a-EV secretion. Coatomer was identified as the EV-binding protein for the EBP. Analysis of cocrystal structures, binding thermodynamics, and mutagenesis found that a dilysine motif mediates EBP binding to coatomer with a conserved function across the Wnt family. We showed that EBP is required for Wnt7a bioactivity when expressed in vivo during regeneration. Overall, our study has elucidated the structural basis and singularity of Wnt secretion on EVs, alternatively to canonical secretion, opening avenues for innovative therapeutic targeting strategies and systemic protein delivery.

## Teaser

A signal peptide that directs Wnt secretion on EVs was identified, and the structural mechanism for this secretion elucidated.

## INTRODUCTION

Wnt proteins are a conserved family of secreted glycoproteins that govern essential developmental, growth, and regenerative processes and are also involved in pathological conditions such as cancer (*1*, *2*). Wnt signaling plays multiple roles in regulating stem cell function, including proliferation, cell polarity and symmetric division, motility, and fate specification (*3*, *4*). Despite the relative hydrophobicity due to palmitoylation (*5*), Wnt proteins actively participate in long-range paracrine signaling between Wnt-producing cells and distal recipient cells (*6*). Several mechanisms have been proposed to explain long-range Wnt signaling including transfer of Wnt proteins via lipoproteins (*7*, *8*), cell extensions called cytonemes (*9*), association with soluble Wnt-binding proteins (*10*, *11*), or via small extracellular vesicles (EVs) called exosomes (*12*–*14*).

Exosomes are small 40- to 150-nm EVs of endocytic origin involved in intercellular communication that transfer different bioactive cargo such as lipids, proteins, microRNAs, and mRNAs to distal cells (*15*). Several in vitro studies have shown that different Wnt proteins are secreted on the surface of exosomal EVs and that EV-Wnts are fully capable of eliciting appropriate signaling in target cells (*12*–*14*). Considerable in vivo evidence derived from studies in *Caenorhabditis* (*16*) and *Drosophila* (*12*, *17*, *18*) support the importance of EVs for long-range Wnt signaling, yet how Wnts are tethered to these migratory organelles remains unknown.

Following acute injury in adult skeletal muscle, Wnt7a is highly up-regulated where it positively stimulates regenerative myogenesis by acting at multiple levels (*19*). Wnt7a/Fzd7 signaling via the planar cell polarity pathway stimulates symmetric muscle stem cell expansion and motility (*20*). Wnt7a/Fzd7 signaling via the AKT/mTOR pathway in myofibers stimulates anabolic growth and hypertrophy (*21*). Consequently, intramuscular injection of Wnt7a protein significantly ameliorates disease progression in *mdx* mice, a mouse model for Duchenne muscular dystrophy (DMD) (*22*). Together, these findings indicate that Wnt7a is a promising candidate therapy for DMD. However, systemic delivery of Wnt7a via the circulation has remained a challenge because of the high hydrophobicity conferred by the conserved palmitoylation (*23*). We therefore set out to determine whether Wnt7a is secreted on EVs and to decipher the mechanism by which Wnt7a is loaded onto EVs to provide insight into long-range Wnt signaling for the treatment of neuromuscular diseases.

We performed structure-function deletion analysis and identified a 18–amino acid signal sequence in Wnt7a that we termed the exosome-binding peptide (EBP). Notably, linking of the EBP sequence to other proteins resulted in their secretion on EVs. Unexpectedly, we found that palmitoylation or the N-terminal signal peptide (SP) was not required for Wnt7a secretion on EVs. WLS knockdown abrogated non-EV bound Wnt7a secretion but only partially diminished Wnt7a-EV secretion. Using BioID, we identified coatomer proteins, COPA and COPB2, as necessary for EBP binding and localization of Wnt7a to the exterior of EVs. Last, structural analysis of cocrystals, isothermal titration calorimetry (ITC), and mutagenesis revealed that direct binding occurs between COPB2 and the positively charged dilysine motif KIK within the EBP. Ultimately, we have found that disruption of Wnt7a interaction with coatomer impairs the in vivo myoregenerative response. We provide evidence that this mechanism appears conserved across the Wnt family. Our findings elucidated a noncanonical structural mechanism that mediates Wnt secretion on EVs and provides insight into the singularity of long-range Wnt-EV signaling.

## RESULTS

### Wnt7a is secreted on EVs following muscle injury

**Fig. 1.**
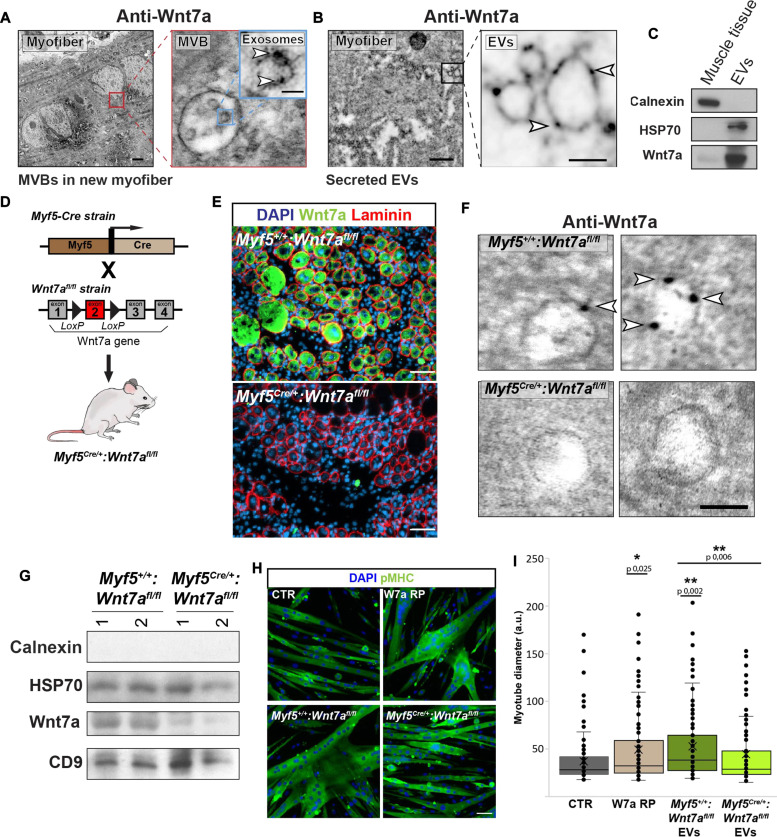
Muscle injury triggers secretion of Wnt7a on the surface of EVs. (**A**) iTEM of anti-Wnt7a labeling of new regenerating myofibers at 96 hours after CTX injury from wild-type (WT) mice shows Wnt7a secretion on ILVs contained in a MVB. Scale bars, 500 and 100 nm. (**B**) iTEM of anti-Wnt7a labeling of injured TA muscle from WT mice shows the presence of Wnt7a on EVs infiltrated on the injured tissue that surrounds the new regenerating myofibers. Scale bar, 500 and 100 nm. (**C**) Immunoblot analysis of EVs fraction from muscle showing Wnt7a expression. (**D**) Schematic representation of mouse strains used to generate conditional Wnt7a floxed in Myf5-expressing cells. (**E**) Immunofluorescence confirmation of Wnt7a expression abrogation in *Myf5^(Cre/+)^:Wnt7a^(fl/fl)^* injured TA at 96 hours after CTX injury. Scale bar, 50 μm. (**F**) iTEM of anti-Wnt7a labeling of EVs showing abrogation of Wnt7a expression in EVs from *Myf5^(Cre/+)^: Wnt7a^(fl/fl)^* mice muscle explants. Scale bar, 100 nm. (**G**) Immunoblot verification of Wnt7a expression abrogation in EVs isolated *from Myf5^(Cre/+)^: Wnt7a^(fl/fl)^* hindlimb muscle at 96 hours after CTX injury. (**H**) pMHC immunofluorescence representative images of hypertrophied myotubes after muscle EVs stimulation containing Wnt7a (*n* = 3). Scale bar, 50 μm. Experiments are representative of *n* = 4 biological replicates. (**I**) Hypertrophy assay of murine primary myotubes treated with EVs from muscle decreases hypertrophy after Wnt7a deletion. Wnt7a recombinant protein was used as a positive control. *n* = 3 mice each with eight technical replicates, means ± SEM. *P* values were determined by two-sided Student’s *t* test (*P < 0.05, **P < 0.005, and ***P < 0.0005). iTEM, immunogold transmission electron microscopy; MVBs, multivesicular bodies; pMHC, pan myosin heavy chain.

Wnt7a expression is highly upregulated in newly differentiating myofibers following acute injury of skeletal muscle (*24*). Examination of muscle cryosections 96 hours following cardiotoxin (CTX) injury by immunogold electron transmission microscopy (iTEM) labeling revealed that regenerating myofibers generate numerous multivesicular bodies (MVBs) that contain intraluminal vesicles (ILVs) with membrane associated Wnt7a (Wnt7a-EV) (Fig. 1A). Notably, the surrounding injured tissue was highly infiltrated with Wnt7a-EVs, presumably EVs secreted by the regenerating myofibers (Fig. 1B). We did not detect Wnt7a not associated with EVs.

We next ask whether Wnt7a-EVs isolated from regenerating muscle were capable of activating appropriate signaling in myogenic cells. Therefore, EVs were isolated from regenerating muscle using a modified tangential flow filtration (TFF) protocol (fig. S1A) (*25*). EVs isolated from regenerating muscle displayed the expected size distribution (fig. S1B) and carried high levels of Wnt7a (Fig. 1C). Wnt7a-EVs isolated from regenerating muscle readily induced hypertrophy of cultured myotubes (fig. S1, C and D), confirming robust bioactivity of Wnt7a-EVs.

To confirm that the bioactivity of Wnt7a-EVs was attributable to Wnt7a, EVs were then isolated by TFF from regenerating muscle from mice with a functional Wnt7a gene (*Myf5^+/+^:Wnt7a^fl/fl^*) (*26*) or from mice where Wnt7a is specifically deleted in muscle (*Myf5^Cre/+^:Wnt7a^fl/fl^*) (Fig. 1D). Immunofluorescence from injured *Myf5^Cre/+^:Wnt7a^fl/fl^* muscle indicates an absence of Wnt7a, whereas injured *Myf5^+/+^:Wnt7a^fl/fl^* muscle exhibits high-level expression of Wnt7a (Fig. 1E). Accordingly, iTEM and immunoblot analysis of EVs isolated from injured *Myf5^Cre/+^:Wnt7a^fl/fl^* muscle revealed an absence of Wnt7a, whereas EVs isolated from injured *Myf5^+/+^:Wnt7a^fl/fl^* muscle exhibit the presence of high levels of Wnt7a (Fig. 1, F and G, and fig. S1E). Note the presence of a background band with higher mobility in Western blots in the absence of Wnt7a (Fig. 1G).

EVs isolated from Wnt7a expressing regenerating *Myf5^+/+^:Wnt7a^fl/fl^* muscle induced a hypertrophic response in primary murine myotubes in a similar manner to recombinant Wnt7a (Fig. 1, H and I). By contrast, EVs isolated from *Myf5^Cre/+^:Wnt7a^fl/fl^* regenerating muscle lacking Wnt7a did not induce significant hypertrophy (Fig. 1, H and I). Therefore, our iTEM and further validated in vitro data suggests that Wnt7a secreted on EVs upon a muscle injury has an active role in the regenerative muscle response in vivo.

### Wnt7a secretion on EVs is regulated by an internal sequence

To facilitate molecular and biochemical analysis of the mechanisms that mediate Wnt7a secretion on EVs, we transfected plasmids expressing Wnt7a into human embryonic kidney HEK293T cells. Quantification following TFF purification indicates that more than 60% of secreted Wnt7a from HEK293T cells is bound to EVs (fig. S2, A and B). Note, we have not quantified the fraction of the Wnt7a retained in the membrane. Dynamic light scattering measurement and iTEM confirmed that the purified EVs exhibited a size distribution with the highest concentration around 150 nm and no difference in diameter between EVs from empty vector– or Wnt7a-transfected HEK293T cells (fig. S2, C and D).

**Fig. 2.**
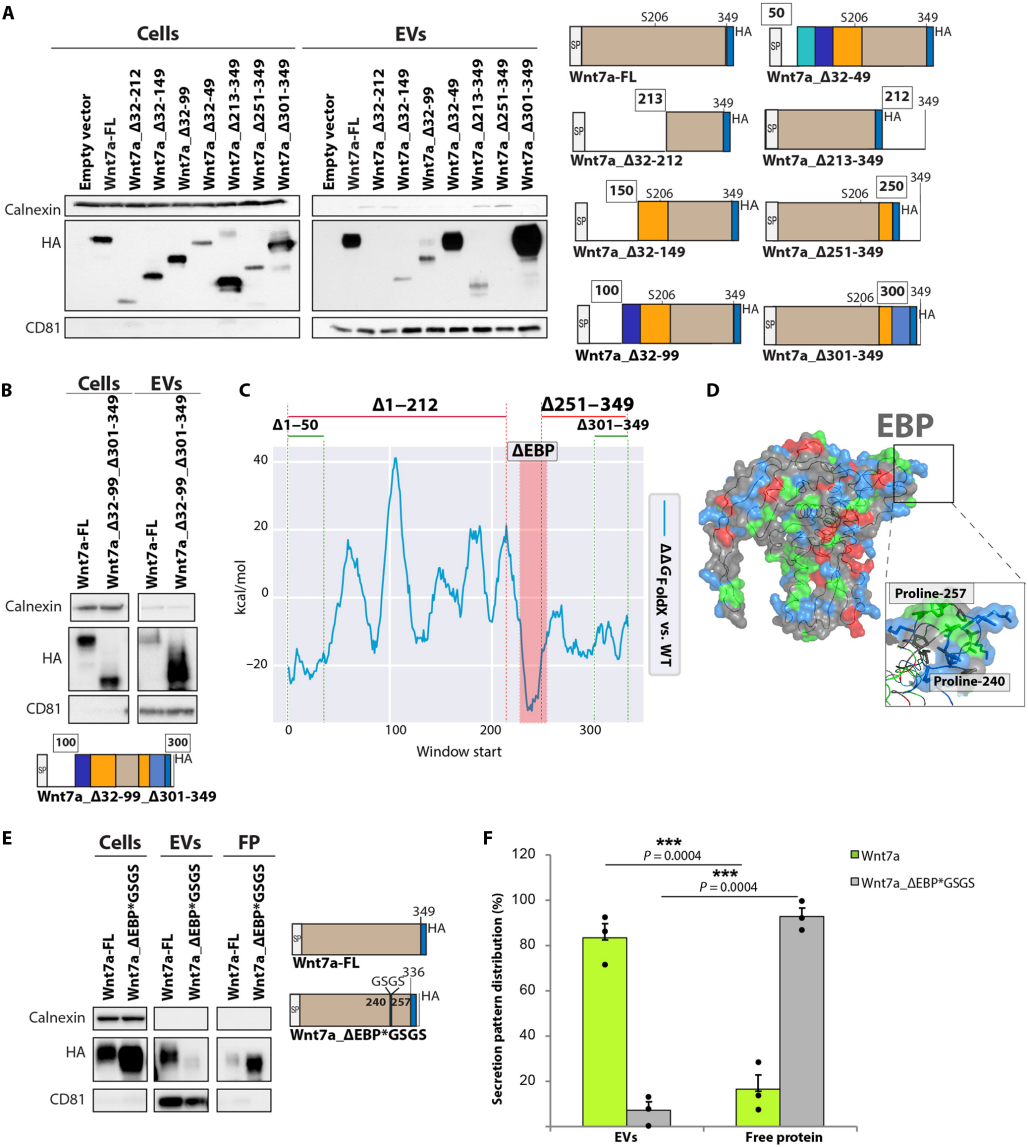
Wnt7a secretion on EVs is regulated by an internal peptide sequence. (**A**) Immunoblot analysis shows interruption of EV secretion following deletion beyond position 100 amino acid but not after position 300. (**B**) Immunoblot analysis showing the internal 100– to 300–amino acid sequence of Wnt7a is sufficient for EV secretion. (**C**) Δ*G*_FoldX_ of Wnt7a indicates Δ1–49 and Δ301–349 do not affect folding—ΔΔ*G* < 0 respect to WT protein—and function is not lost. Δ1–212 affects protein folding. Δ251–349 does not affect folding, but function is lost because a region of the EBP is truncated. (**D**) Surface of Wnt7a with negative charged residues in red, positive charged residues in blue, and hydrophobic residues in green. The EBP is positively charged. (**E**) Immunoblot analysis, demonstrates replacement of EBP with a GSGS linker abrogates EV secretion in favor of non-EV Wnt7a secretion. FP, free protein. (**F**) Quantification of Wnt7a expression on EVs and non-EV protein fractions with and without EBP. Experiments are representative of three independent biological replicates performed in HEK293T cells transfected with different Wnt7a-HA tagged truncates. *n* = 3 biological replicates, means ± SEM. *P* values were determined by two-sided Student’s *t* test (***P < 0.0005). HA, hemagglutinin.

To map the region required for secretion on EVs, we constructed a series of N-terminal and C-terminal deletions of Wnt7a–hemagglutinin (HA) (Fig. 2A). Initially, N-terminal deletions were performed leaving in place the 31–amino acid SP required for secretion of Wnt7a as protein (*2*). The Wnt7a variants were expressed in HEK293T cells, and the amount of Wnt7a secreted on EVs was assessed by immunoblot analysis (Fig. 2A and fig. S2E). Wnt7a secretion on EVs was not impaired upon deletion of the N-terminal 18 amino acids following the SP (Wnt7a_Δ32–49) and the C-terminal 48 amino acids (Wnt7a_Δ301–349) (Fig. 2A). By contrast, the deletion of additional sequences from the N terminus (Wnt7a_Δ32–212) and C-terminus (Wnt7a_Δ251–349) appeared to abrogate secretion on EVs (Fig. 2A and fig. S2E). However, Wnt7a lacking both the first 99 amino acids and the last 48 amino acids (Wnt7a_Δ1-99_Δ301–349) were secreted on EVs (Fig. 2B).

To identify the region that mediates the targeting to EVs, we analyzed a three-dimensional (3D) model of Wnt7a aligned on the structure of XWnt8a (fig. S2F) (*23*). Energetic analysis after truncating successive 15–amino acid residue regions using FoldX (ΔGFoldX) (*27*) revealed that the deletion of amino acids between positions 240 and 257 would be predicted to not interfere with Wnt7a structural folding stability (Fig. 2C). This low energetic region is a result of a hydrophobic random coil structure flanked by two prolines between position 240 and 257 (Fig. 2D).

The region was then investigated as a potential binding site that would mediate targeting of Wnt7a to EVs. Replacement of the 18–amino acid sequence between position 240 and 257 with the linker domain GSGS (Wnt7a_ΔEBP*GSGS) resulted in a loss of Wnt7a targeting to EVs with a corresponding increase in secretion of non-EV Wnt7a protein and with no effect on total Wnt7a protein expression (Fig. 2, E and F). However, it should be noted that this analysis does not include the amount of secreted Wnt7a that remains bound to the exterior surface of the plasma membrane. Therefore, the sequence PVRASRNKRPTFLKIKKP, which we term the EBP, is required for targeting Wnt7a to EVs.

### The EBP is sufficient to confer targeting to EVs

We next investigated whether the EBP is sufficient for targeting proteins for secretion on EVs. We first added the EBP to a truncated Wnt7a that we previously found to not localize to EVs (Wnt7a_Δ213–349) (Fig. 2A). We chose a specific insertion site for the EBP within the Wnt7a_Δ213–349 truncate to avoid any conformational disruption or EBP offshoring. Energetic and conformational studies with FoldX showed a loop starting at position 172 as a potential insertion site for the EBP with a similar distance between loop terminals (~11 Å versus ~10.5 Å) and proximal in the 3D space to the original EBP location (fig. S3A).

**Fig. 3.**
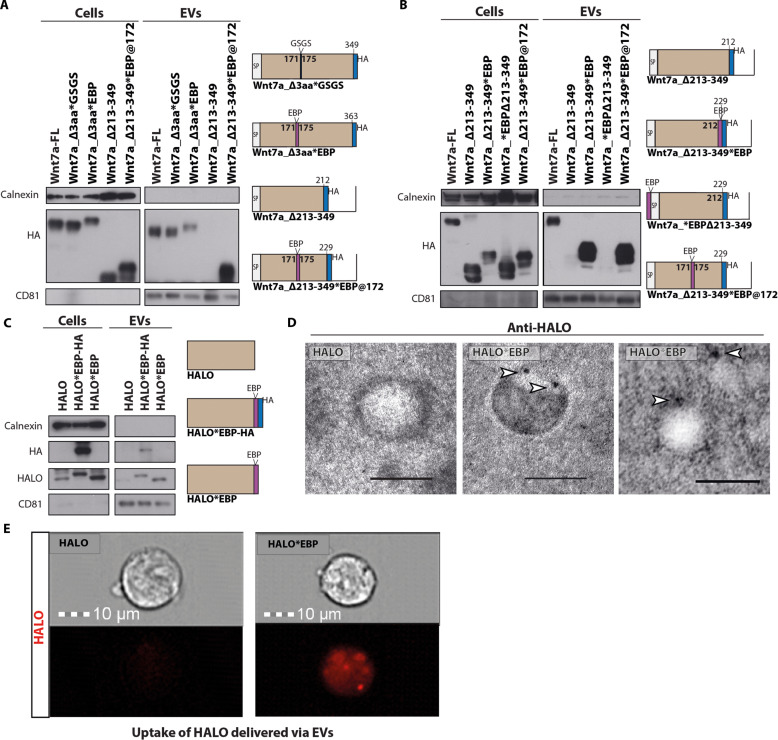
The EBP is sufficient to confer targeting to EVs. (**A**) Restoration of EV secretion by inserting EBP into an upstream domain of Wnt7a truncate that is not secreted on EVs. The insertion does not perturb the stability of the full-length protein (Wnt7a-Δ3aa*GSG versus Wnt7a-Δ3aa*EBP). Insertion of EBP to this site (Wnt7a-Δ213-249*EBP) restores EVs localization to Wnt7a-Δ213-249. (**B**) C-terminal linked EBP but not N-terminal linked confers targeting of Wnt7a-Δ213-249 onto EVs. (**C**) Linking of EBP HALO protein results in EV secretion. (**D**) iTEM images of EVs with anti-HALO immunostaining showing the expression of HALO*EBP on the surface of EVs. Scale bars, 100 μm. (**E**) Detection of HALO by fluorescence inside HEK293T cells following treatment with HALO*EBP EVs versus HALO EVs derived from HEK293T cells transfected with different Wnt7a-HA and HALO tagged truncates. *n* = 3 biological replicates.

Insertion of the linker GSG or EBP between position 171 and 175 into full-length Wnt7a-HA (Wnt7a-FL) had no effect on secretion on EVs (Fig. 3A). Notably, insertion of the EBP between position 171 and 175 into Wnt7a_Δ213–349 (Wnt7a_Δ213–349*EBP@172) fully restored secretion on EVs (Fig. 3A). Moreover, the addition of EBP to the C terminus of Wnt7a_Δ213–349 (Wnt7a_Δ213–349*EBP@212) confirmed the structurally independent capacity of EBP to target proteins for secretion on EVs (Fig. 3B). By contrast, the addition of the EBP to the N terminus of Wnt7a_Δ213–349 adjacent the SP (Wnt7a_*EBPΔ213–349) did not result in secretion on EVs (Fig. 3B), suggesting that proximity of both SPs interferes with EV targeting.

We next asked whether linking the EBP to a non-Wnt protein would direct secretion on EVs. Therefore, the EBP was fused to the HALO tag, a 297–amino acid peptide derived from a bacterial enzyme that can covalently bind fluorescent ligands (Fig. 3C) (*28*). The HALO protein alone was not secreted on EVs, whereas HALO*EBP-HA and HALO*EBP were both efficiently secreted on EVs (Fig. 3C). The detection of HALO using iTEM confirmed the presence of HALO*EBP on the surface of EVs (Fig. 3D). Furthermore, purified EVs efficiently delivered the EBP-tagged HALO protein to recipient HEK293T cells, as assessed by labeling EVs with a specific fluorescent tag for HALO (Fig. 3E and fig. S3, B and C). By contrast, EVs isolated from HALO-overexpressing cells did not deliver HALO to recipient cells as revealed by the absence of fluorescence staining (Fig. 3E). Therefore, we conclude that the EBP is sufficient to mediate targeting of proteins to EVs that can then be delivered to recipient cells.

### Wnt7a-EV secretion does not require the SP or palmitoylation

**Fig. 4.**
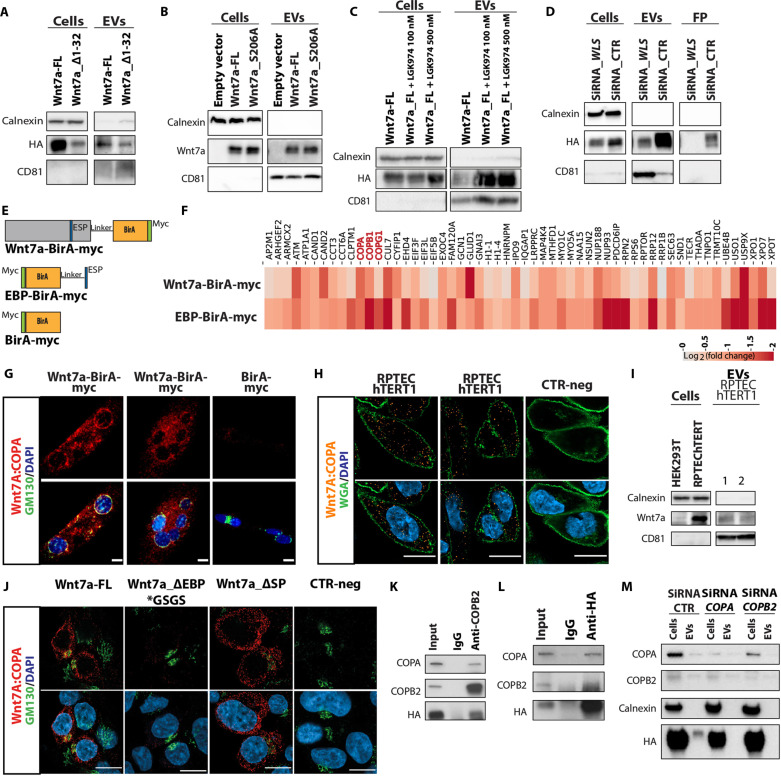
Wnt7a secretion on EVs appears independent of the canonical pathway and requires Coatomer. (**A**) SP is not required for EVs-Wnt7a secretion in transfected HEK293T cells. (**B**) Mutant Wnt7a_S206A, lacking the palmitoylation site, is secreted on EVs in transfected HEK293T cells. (**C**) Drug inhibition of PORCN does not affect secretion of Wnt7a on EVs. (**D**) Knockdown of *WLS* in siRNA partially affects secretion of Wnt7a on EVs but does abolish secretion of non-EV Wnt7a. (**E**) BirA constructs for BioID analysis. (**F**) Heatmap displaying fold change (log_2_ scale) of enriched proteins in mass spectrometry (ESP_BirA:BirA and Wnt7a_BirA:BirA). Shown are enrichment of >50% [log_2_ (FC) > 0.5849] on EBP and a positive enrichment [log_2_ (FC) > 0] on Wnt7a. (**G**) Wnt7a: COPA PLA (red) performed in murine primary myotubes either expressing Wnt7a-BirA or BirA. GM310 in green and 4′,6-diamidino-2-phenylindole (DAPI) in blue. Scale bars, 10 μm. (**H**) Wnt7a:COPA PLA (orange) performed in RPTEC- hTERT1 cells. Wheat Germ Agglutinin (WGA) in green and DAPI in blue. CTR-neg is control without Wnt7a antibody. Scale bars, 10 μm. (**I**) Wnt7a is secreted on EVs derived from RPTEC-hTERT1 cells. HEK293T cells lacking Wnt7a were used as a negative control. (**J**) Wnt7a:COPA PLA (red) performed in HEK293T cells expressing Wnt7a-FL, Wnt7a_ΔEBP*GSGS, or Wnt7a_ΔSP. GM310 in green and DAPI in blue. Scale bars, 10 μm. CTR-neg is control without Wnt7a antibody. (**K**) Wnt7a-HA interacts with COPA and COPB2. HEK293T cells overexpressing Wnt7a-HA were immunoprecipitated with COPB2 antibody or (**L**) immunoprecipitated with HA antibody. (**M**) Immunoblot EVs secretion analysis of Wnt7a after siRNA knockdown of *COPA* and *COPB2* shows disruption of Wnt7a-EV secretion in transfected HEK293T cells. *n* = 3 biological replicates. IgG, immunoglobulin G; WGA, Wheat Germ Agglutinin.

To investigate the involvement of the canonical ER-Golgi secretory pathway, we assessed the requirement of several mechanisms that have been attributed to Wnt protein secretion. We found that deletion of the N-terminal SP did not alter the ability of Wnt7a to be secreted on EVs (Fig. 4A). Furthermore, the deletion of the SP from the minimal Wnt7a_Δ1-99_Δ301–349 construct did not affect secretion on EVs (fig. S4A). Notably, Wnt7a_Δ1-99_Δ301–349 showed more than 100% increase of secretion on EVs relative to Wnt7a_Δ32-99_Δ301–349 with the SP (fig. S4B). Last, all versions of Wnt7a that were previously tested (Fig. 2A) are fully secreted on EVs in the absence of SP (fig. S4C).

Several publications have asserted the importance of palmitoylation of Wnt for secretion and activity (*29*–*31*). Therefore, we tested whether Wnt7a was secreted on EVs following mutation of the palmitoylation site at serine-206 (*23*). Previously, we found that palmitoylation is not required for Wnt7a secretion or activity (*32*). Notably, we observed that secretion of Wnt7a on EVs was unaffected by mutation of the palmitoylation site (Fig. 4B). Similarly, inhibition with LGK974, a chemical inhibitor of PORCN (*33*), had no apparent effect on Wnt7a secretion on EVs (Fig. 4C).

We also tested whether WLS is required for Wnt7a secretion on EVs. WLS is a chaperone transmembrane protein that binds Wnt through its palmitoylation site to direct Wnt canonical secretion to the plasma membrane (*34*–*36*). Our data showed that the secretion of non-EV Wnt7a was entirely abolished upon small interfering RNA (siRNA) knockdown of *WLS* (Fig. 4D and fig. S4D). By contrast, Wnt7a secretion on EVs was readily detectable following siRNA knockdown of *WLS* (Fig. 4D). However, we observed a 48% decrease in Wnt7a secretion on EVs upon *WLS* siRNA compared to CTR siRNA, together with a 40% increase in CD81 expression in following the knockdown of *WLS* (Fig. 4D). CD81 levels are up-regulated in the ectosomes relative to exosomes (*37*). Unfortunately, current isolation methodologies for EVs cannot distinguish between ectosomes and exosomes. Therefore, we speculate that the increase in CD81 levels is a consequence of an increase in the proportion of ectosomes. Thus, Wnt7a exosomal secretion is diminished, but further research is required to discern the role of WLS in Wnt7a-EVs secretion in ectosomes, exosomes, and non-EV–related secretion.

### Wnt7a binds coatomer proteins

To elucidate the molecular basis whereby the EBP mediates the secretion of Wnt7a on EVs, we performed BioID interactome analysis to identify potential EV-binding proteins using BirA (*38*), a biotin ligase that biotinylates proteins in close proximity to the bait protein (fig. S4E). Mouse primary myoblasts were generated to express myc-tagged Wnt7a-BirA, EBP-BirA, or BirA (Fig. 4E and fig. S4F). In addition, the ability of Wnt7a-BirA-myc to be localized to myoblasts-derived EVs was verified by mass spectrometry (fig. S4G). Moreover, iTEM revealed localization of Wnt7a and the Myc tag on the EV surface regardless of permeabilization (fig. S4, H and I).

Mass spectrometric identification of biotinylated proteins isolated from transfected primary myoblasts revealed that coatomer proteins COPA and COPB2 were among the most enriched candidates within the EBP-BirA and Wnt7a-BirA interactomes (Fig. 4F). Gene Ontology term analysis of the interactome indicated that coatomer proteins were among the highest represented hits (fig. S4J). COPA and COPB2 have been previously noted to be present at high levels on EVs (*39*, *40*).

Proximity ligation assays (PLA) between Wnt7a and COPA or COPB2 in myotubes from murine primary myoblast, confirmed the interaction of Wnt7a with these Coatomer components (Fig. 4G and fig. S4K, respectively). PLA detected the interaction between Wnt7a and COPA primarily at the Golgi, whereas the interaction between Wnt7a and COPB2 appeared proximal to the cell membrane (Fig. 4G and fig. S4K, respectively), consistent with the suggestion that different coatomer proteins have distinct roles in endosomes and MVBs as previously described (*41*–*43*).

These findings were corroborated in a human epithelial immortalized RPTEC-hTERT1 cells derived from the kidney cortex that endogenously express Wnt7a (fig. S4, L and M). PLA between Wnt7a and COPA revealed the interaction of endogenous Wnt7a with COPA in the cytoplasm (Fig. 4H). Immunoblot analysis demonstrates that RPTEC-hTERT1 cells secrete Wnt7a on EVs (Fig. 4I). Therefore, the interaction of Wnt7a with Coatomer and secretion on EVs is not a consequence of overexpression.

These results were also verified in HEK293T cells. PLA did not detect an interaction between Wnt7a_ ΔEBP*GSGS (lacking the EBP) and COPA, whereas the interaction was detected between Wnt7a_ ΔSP (lacking the N-terminal SP) and COPA (Fig. 4J and fig. S4N). Reciprocal immunoprecipitation–Western blot analysis suggested that Wnt7a interacts with COPA and COPB2 in the same complex (Fig. 4, K and L). Mass spectrometry analysis of anti-HA antibody immunoprecipitates from Wnt7a-HA–expressing EVs confirmed the interaction of COPA and COPB2 with Wnt7a in EVs (fig. S4O).

To assess the requirement of COPA and COPB2 for Wnt7a secretion on EVs, siRNA knockdown of *COPA* and *COPB2* was performed, which resulted in an abrogation of Wnt7a secretion on EVs (Fig. 4M). Together, these results suggest an alternative secretion mechanism whereby Wnt7a trafficking to EVs occurs through an interaction with the coatomer proteins.

### The KxK motif mediates EBP binding to coatomer

**Fig. 5.**
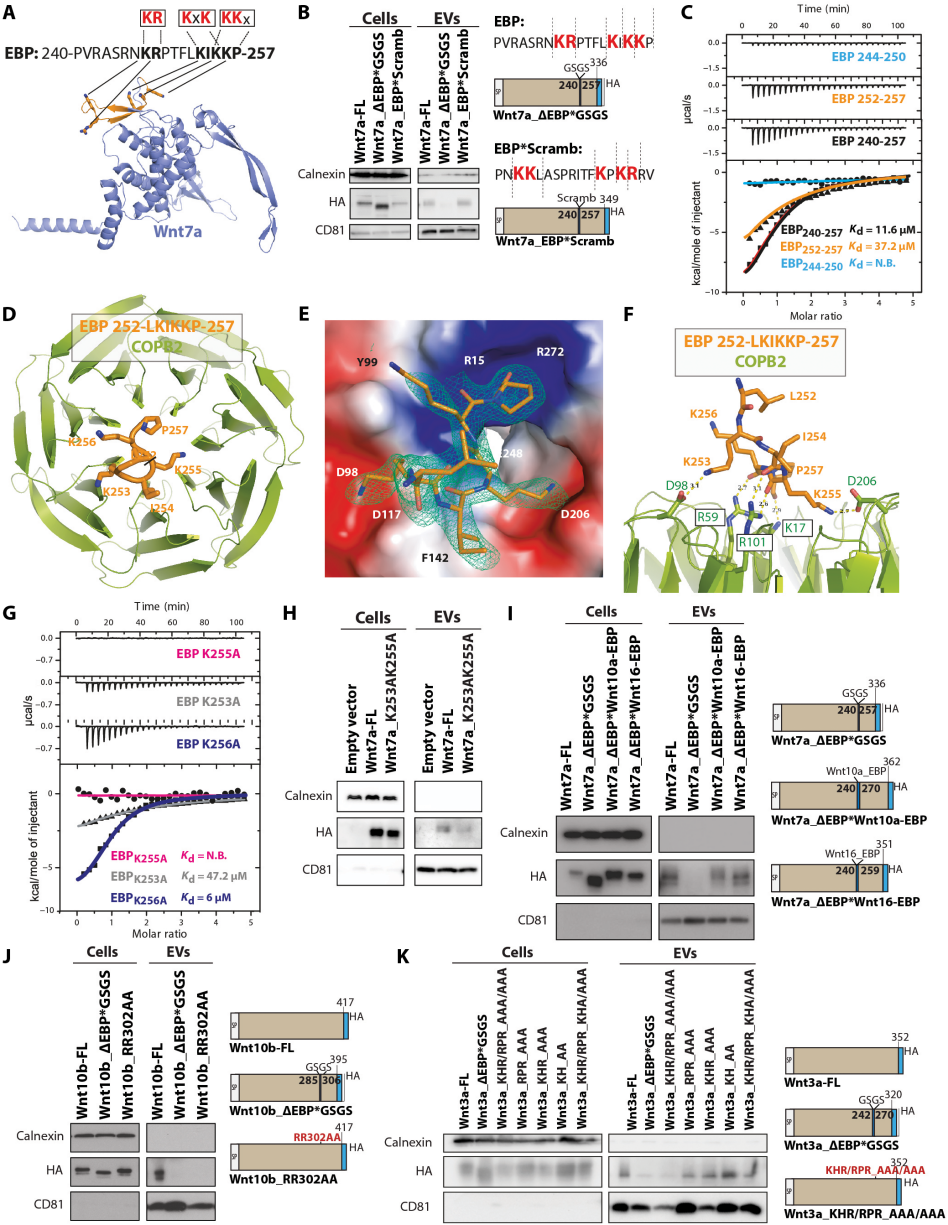
Wnt7a binds Coatomer proteins. (**A**) Predicted Wnt7a structure in AlphaFold (blue) with the EBP region highlighted in orange and the three positively charged motifs (red) within the EBP. Note that EBP is a solvent-exposed region. (**B**) Wnt7a-ESP*Scramble mutant maintains the dilysine motif and exhibits no impairment in EV secretion. (**C**) ITC measurements for COPB2_1–304_ binding to potential dilysine/arginine motifs within the EBP. WT COPB2_1–304_ binds to the LKIKKP subregion. (**D** to **F**) views of the KxKx motif of Wnt7a bound to COPB2_1–304_. (D) Top view of the WD-repeat domain of COPB_1–304_ (green) with the LKIKKP peptide (orange) in ribbon representation. (E) Close-up view of the LKIKKP peptide with a difference electron density map calculated by omitting the peptide and contoured at 3σ (blue mesh). COPB2 surface is colored by electrostatic potential ranging from −5 kT/e (red) to 5 kT/e (blue). (F) Lateral view of the binding motif with hydrogen bonds and distances. (**G**) Structure-based point mutations confirm the molecular recognition of the KxKx motif in ITC assays. (**H**) Double lysine mutation of K253 and K255 by alanine disrupts Wnt7a-EV secretion. (**I**) Replacement of Wnt7a-EBP by either Wnt10a-EBP or Wnt16 EBP containing KR and RR (right). Replacement with Wnt10a-EBP or Wnt16 EBP rescues Wnt7a-EV secretion. (**J**) EV secretion analysis of Wnt10b after EBP removal or double arginine mutation within its EBP (right). Double arginine mutation disrupts secretion of Wnt10b on EVs to the same extent as removal of the entire Wnt10b EBP sequence. (**K**) Secretion analysis of Wnt3a after EBP removal or mutation of the entire positively charged motifs RPR, KHR, and KH within its EBP (right). Only concomitant mutation of RPR and KHR motifs disrupts secretion of Wnt3a on EVS to the same extent as removal of the entire Wnt3a EBP sequence. *n* = 3 biologic replicates.

Polypeptides containing the positively charged motifs KKxx, KxKxx, or RKxx have been previously shown to bind COPA and COPB2 (*44*). Therefore, we evaluated whether the KR, KIK, and KKP motifs within the EBP are required for secretion of Wnt7a on EVs (Fig. 5A). Notably, replacing the EBP with a scrambled sequence (PN**KKL**ASPRITF**KPKR**RV), which maintains the positively charged motifs (Wnt7a_EBP*Scramb), had no effect on Wnt7a-EV secretion in HEK293T cells (Fig. 5B).

Isothermal titration calorimetry (ITC) confirmed high affinity binding of the 18–amino acid EBP polypeptide with the N-terminal WD repeat of COPB2 with a dissociation constant (*K*_d_) of 11.6 μM (Fig. 5C). The C-terminal half of the polypeptide (252 to 257) is bound to COPB2 with a *K*_d_ of 37.2 μM. By contrast, the N-terminal half (244 to 250) did not bind to COPB2. These data support the notion that the KIKK motif mediates binding to COPB2 (Fig. 5C and table S1).

To elucidate the molecular details for the interaction, we solved the structure of the EBP (252 to 257) bound to the COPB2 at 1.8-Å resolution by x-ray crystallography (Fig. 5, D to F, and table S2). The interaction involves two main contacts of the lysine side chains K253 and K255 with two acidic patches at the central opening of the β-propeller top face (Fig. 5E). More specifically, the amino groups of K253 and K255 interact with the carboxylate groups of D98 and D206 within the WD repeat domain (*Saccharomyces cerevisiae* COPB2 numbering) (Fig. 5F). In addition to the two primary contacts, a secondary contact involves the backbone carbonyl oxygen atoms K255, K256, and P257 of EBP, which interact with the guanidinium groups of R59 and R101 and the amino group of K17 in the WD repeat domain (Fig. 5F). The binding mode of EBP to COPB2 is very similar to that of the KxKxx motif of yeast Emp47p, with sequence IKTKLL (fig. S5A) and with comparable affinity (*K*_d_ = 49 μM for Emp47p) (*45*).

We tested our model in solution using a combination of ITC and structure-guided mutants. As expected, K255A or K253A substitutions markedly reduced or even abolished the EBP-COPB2 interaction, whereas K256A substitution, which is not involved in COPB2 binding, did not substantially affect the affinity (Fig. 5G). To confirm the ITC results, we transfected HEK293T cells with a double-point mutation of the lysine residues to alanine K253 and K255 (Fig. 5H). Immunoblot data corroborated that disruption of the KIK motif reduces the secretion of Wnt7a on EVs. These data together confirm that COPB2 and COPA regulate Wnt7a trafficking to EVs by interacting with the KIK motif within the EBP (Fig. 5H).

The EBP appears to function as a linking peptide that connects the N- and C-terminal domains of the 19 human Wnt proteins with a sequence that is highly variable in length and amino acid composition (fig. S5B). Notably, the KK motif is present in Wnt2, and other positively charged motifs, such as KR, are present in Wnt5b, Wnt8a, Wnt11, and Wnt16 (fig. S5B). Moreover, another positively charged motif, RR, is present in Wnt2b, Wnt4, Wnt10a, Wnt10b, and Wnt16 proteins (fig. S5B). Together, this suggests the possibility of a conserved mechanism mediating secretion on EVs across the entire Wnt family.

To test the ability of candidate EBPs from different Wnts to mediate secretion on EVs, we transfected HEK293T cells with a mutant where the EBP of Wnt7a was replaced with either the EBP from Wnt10a, containing only the RR motif, or the EBP from Wnt16, containing both motifs RR and KR (fig. S5B). Both the Wnt10a and the Wnt16 EBP chimeras were efficiently secreted on EVs (Fig. 5I). Furthermore, the deletion of the EBP from Wnt10b, or double mutation of its RR motif, completely abrogated secretion of Wnt10b on EVs (Fig. 5J). Similar results were obtained in HEK293T cells with Wnt3a that contains three positively charged motifs RPR, KHR, and KH. Point mutations of these three motifs separately showed no abrogation of Wnt3a secretion (Fig. 5K). Conversely, mutation of the three motifs together resulted in the loss of Wnt3a secretion on EVs, as does the deletion of the entire EBP in Wnt3a (Fig. 5K).

The Wnt exosomal secretory mechanism appears to be evolutionarily well conserved. Wg, the Wnt ortholog in *Drosophila Melanogaster*, contains a similar disordered loop in the linker region that contains KR and KK motifs (fig. S5C). Moreover, Drosophila Wg has been found to be secreted on EVs (*12*, *17*, *18*). In addition, the Wnt7a EBP region is highly conserved across evolution (fig. S5D). Together, these results suggest that the direct binding of coatomer with Wnt family members via positively charged motifs, within the EBP domain, represents a conserved mechanism that mediates the secretion and localization of Wnts on the surface of EVs.

### Wnt7a interaction with coatomer is required for the muscle regenerative response

**Fig. 6.**
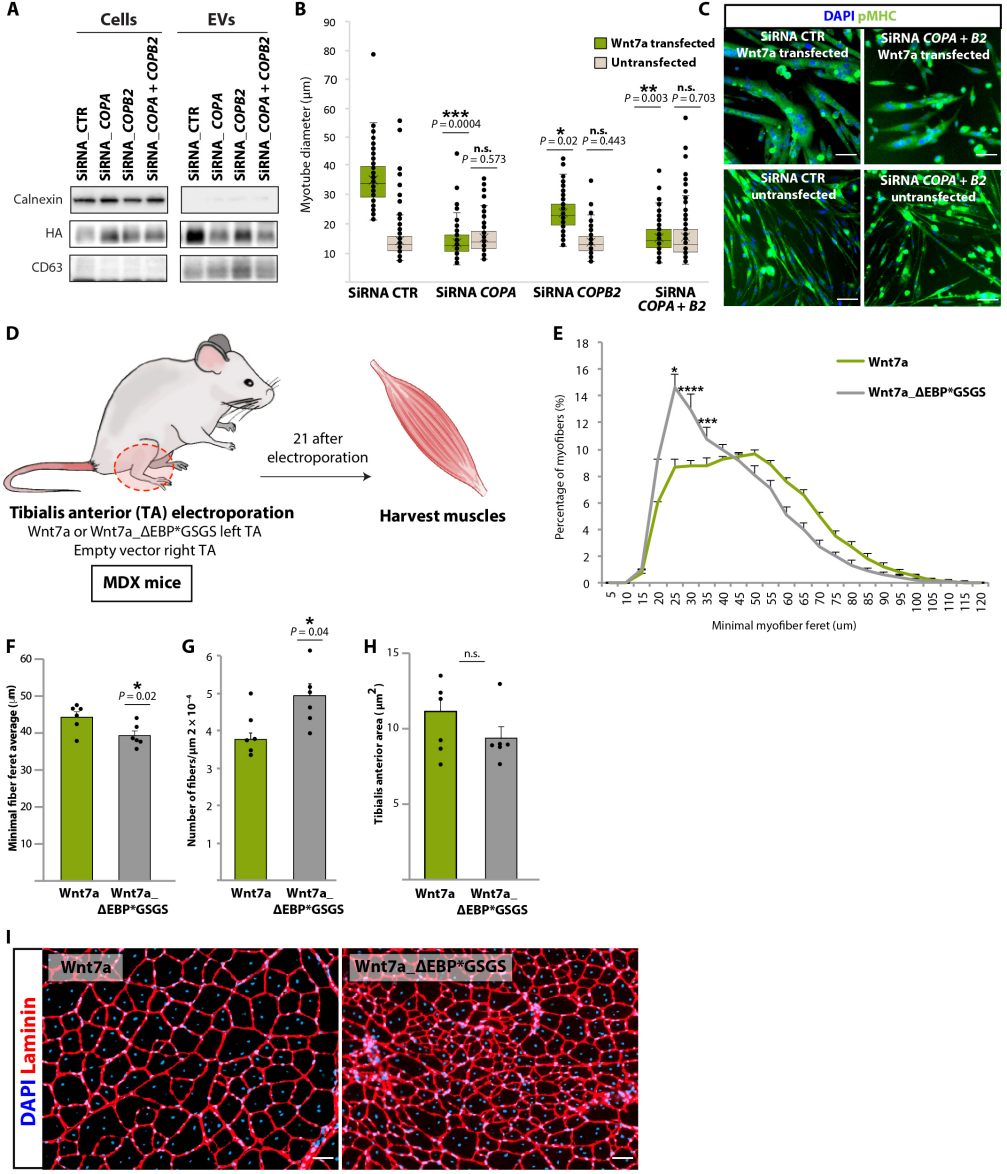
Wnt7a interaction with Coatomer is required for the muscle regenerative response. (**A**) Knockdown of *COPA* and *COPB2* using siRNA disrupts Wnt7a-EV secretion in Wnt7a stably expressing primary myoblasts. (**B**) Knockdown of *COPA* and *COPB2* decreases hypertrophy in Wnt7a-transfected myotubes. Conversely, knockdown did not affect hypertrophy in myotubes not expressing Wnt7a. (**C**) Representative images after simultaneously siRNA knockdown of *COPA* and *COPB2* in myotubes. Scale bars, 50 μm. (**D**) Schematic representation of in vivo workflow. (**E**) Myofiber caliber distribution comparing TA electroporated with Wnt7a (green) versus Wnt7a_ΔEBP*GSGS (gray). (**F**) Average minimal fiber feret comparing TA electroporated with Wnt7a (green) versus Wnt7a_ΔEBP*GSGS (gray). (**G**) Quantification of fiber number comparing TA electroporated with Wnt7a (green) versus Wnt7a_ΔEBP*GSGS (gray). (**H**) Quantification of muscle area comparing TA electroporated with Wnt7a (green) versus Wnt7a_ΔEBP*GSGS (gray). (**I**) Section of TA muscles showing reduced myofiber caliber an increased number of myofibers after electroporation of Wnt7a_ΔEBP*GSGS into TA muscle of *mdx* mice. Scale bars, 100 μm. TA, tibialis anterior. In vitro experiments are representative of three independent biological replicates performed in murine primary myoblasts. In vivo experiments are representative *n* = 6 mice, means ± SEM. *P* value was determined by two-sided Student’s *t* test (*P < 0.05 and **P < 0.005).

To investigate the role of Wnt7a-Coatomer interaction during the muscle regeneration, we first tested Wnt7a secretion on EVs in cultured primary myotubes following siRNA-mediated knockdown of *COPA* or *COPB2* (Fig. 6, A to C, and fig. S6A). We observed reduced Wnt7a secretion on EVs following knockdown of *COPA* or *COPB2* (Fig. 6A). Correspondingly, *COPA* and *COPB2* knockdown in Wnt7a-expressing primary myotubes abrogated the hypertrophy response induced by Wnt7a (Fig. 6, B and C). No reduction in myotube diameter was observed following *COPA* or *COPB2* knockdown in nontransfected myotubes that do not express Wnt7a (Fig. 6, B and C). No additive result was found when both *COPA* and *COPB2* were knocked down, suggesting that both proteins are required for Wnt7a secretion on EVs (Fig. 6, B and C).

To assess the requirement for the binding of Wnt7a with Coatomer for muscle regeneration in vivo, we electroporated the Tibialis Anterior (TA) muscle of male *mdx* mice, a mouse model of DMD, with vectors expressing either full-length Wnt7a (Wnt7a-FL) and control vector in the contralateral leg or Wnt7a with the EBP replaced with a GSGS linker (Wnt7a_ΔEBP*GSGS) and control vector in the contralateral (Fig. 6D) and analyzed after 21 days. Previously, we found that expression of Wnt7a in the TA muscle of *mdx* mice significantly increased myofiber caliber (*22*). Electroporation of the Wnt7a-FL expression vector induced the formation of larger caliber myofibers, whereas electroporation of vectors expressing Wnt7a_ΔEBP*GSGS failed to stimulate hypertrophy together with an increase in numbers of myofibers that were small in caliber (Fig. 6, E to I, and fig. S6B). Likewise, the TA analysis intra mice comparing Wnt7a or Wnt7a_ΔEBP*GSGS with its contralateral control leg is shown in fig S6 (C to L). Last, we tested the ability of conditioned media derived from HEK293T transfected with expression vectors for FL_Wnt7a or Wnt7a_ΔEBP*GSGS to induce myotube hypertrophy in vitro. We observed that both forms of Wnt7a induced a similar degree of hypertrophy (fig. S6M). We interpret these results to indicate that while both Wnt7a full length and Wnt7a_ΔEBP*GSGS are similarly active when secreted as non-EV Wnt or EV-Wnt, the inability of Wnt7a_ΔEBP*GSGS to be secreted on EVs in vivo compromises its long-range activity. Together, these data indicate that Wnt7a secretion on EVs is required for Wnt7a bioactivity in vivo during regenerative myogenesis.

## DISCUSSION

We have identified a signal sequence in Wnt7a that we term the EBP that is necessary and sufficient to localize proteins to the surface of EVs for distal secretion facilitating muscle regeneration. We identified the structural basis for binding of the EBP to the Coatomer proteins COPA and COPB2. ITC and mutagenesis confirmed that the direct interaction occurs between COPB2 and the positively charged motif KIK located within the EBP. These findings elucidated the structural mechanism for Wnt secretion on EVs and identified a noncanonical trafficking route that appears to function independently of the classical Wnt secretion routes.

Coatomer proteins have been implicated in noncanonical roles beyond retrograde transport of proteins from Golgi to ER. Coatomer proteins have been shown to be involved in endosomal trafficking and biogenesis of MVBs (*43*) and being highly enriched in EVs (*40*). Wnt5a directs the assembly of the Wnt-receptor-actin-myosin-polarity structure involved in motility and associates with MVBs and coatomer proteins (*46*, *47*). Moreover, the down-regulation of COPB2 by microRNA-4461 inhibits tumorigenesis derived by EVs in colorectal cancer (*48*). Last, it has recently been described how coatomer proteins direct mTOR from the endoplasmic reticulum (ER) to the lysomal membrane (*49*)*.* Therefore, coatomer proteins may be implicated in additional cell functions and pathologies other than coating COPI vesicles for retrograde transport.

The 18–amino acid EBP region has been implicated in other Wnt7a functions. For example, the Reck receptor binds Wnt7a through sequences in the EBP to form a signalosome that induces canonical Wnt7a signaling (*50*, *51*). Notably, we did not detect Reck in our BioID assays, and none of the six critical amino acids within the linker region that bind Reck are involved in the Wnt7a-Coatomer interaction. In addition, WntD and Wnt3a have been shown to bind to LRP6 through the EBP sequence, and this interaction is required to activate canonical ß-catenin–dependent Wnt signaling (*52*). However, not all Wnts require LRP6 for signaling. We previously reported that Wnt7a-Fzd7 signaling in myogenic cells is noncanonical and independent of LRP6 (*53*). Together, these findings suggest that this unstructured loop might act as an intrinsically disordered protein sequence (*54*) to coordinate distinct cell type–specific functions.

Several groups have shown that Wnt secretion requires an interaction with WLS, a chaperone transmembrane protein that facilitates the secretion to the membrane (*34*, *35*). Moreover, WLS interacts with coatomer within COPI vesicles to mediate the recycling of WLS and thus promoting Wnt secretion (*55*). These data can be interpreted to suggest that WLS acts as the linker between coatomer and Wnt facilitating the transfer to the membrane. However, it was recently shown that the interaction between Wnt and WLS is through three hairpins where Wnts are palmitoylated and does not involve the EBP (*36*). In addition, we found that knockdown of *WLS* did not fully block Wnt7a-EV secretion but did block non-EV Wnt7a secretion. Last, we did not detect any interaction of Wnt7a with WLS by BioID.

We and others have published studies that show that non-palmitoylated Wnts are secreted and functional. We previously reported that the C-terminal 134 amino acid of Wnt7a, lacking the putative palmitoylation sites, is efficiently secreted, binds Fzd7, and exhibits full bioactivity (*32*). Strikingly, a non-palmitoylated 34 polypeptide of Wnt7a corresponding to the C-terminal hairpin region bound Fzd7 and was fully biologically active in inducing signalling (*56*). This finding is consistent with cocrystal structural analysis showing that the C-terminal hairpin region of xWnt8 mediates sequence-specific high-affinity binding to Fzd8 (*23*). Here, we show that inhibition of palmitoylation with LGK974 did not block Wnt7a secretion on EVs, but it increased it. In support of these findings, Nusse and coworkers demonstrated that WntD does not require palmitoylated for secretion or activity (*57*). Moreover, neither knockdown nor drug inhibition of porcupine of human astrocytes or in human CD8 T cells had an effect on the endogenously expression or activity of Wnt1, Wnt3, Wnt5b, and Wnt10b or Wnt1, Wnt3, Wnt6, Wnt7a, Wnt10a, and Wnt16, respectively (*58*). They did observe that Wnt2b required palmitoylation for secretion by human primary astrocytes. These findings concur with mass spectrometry analyses where secreted Wnt was observed to lack palmitoylation (*29*). These published reports together with our data provide compelling evidence that palmitoylation is not a prerequisite for Wnt7a secretion or activity. However, it remains entirely possible that palmitoylation is important for other Wnts. It was recently reported that palmitoylation of PDL1 prevents its ESCRT-mediated sorting to MVBs (*59*). Therefore, together, these findings suggest that palmitoylation may represent a candidate mechanism for preventing the secretion of proteins on EVs.

A limitation in our study is that we have not resolved how Wnt7a is trafficked such that it can be tethered to the exterior of EVs. It is possible that proteins that have been secreted via canonical pathways are reinternalized via endocytosis and that these endosomes then mature into late endosomes and then MVBs. Thus, Wnts secreted via the canonical pathway could also be localized to the surface of exosomes. However, our data suggests that transfected Wnt7a does not require an SP for secretion on EVs. Another possibility is direct transport. For example, cytoplasmic proteins have been shown to be imported into lysosomes by a mechanism involving the chaperones Hsp70 or Hspa8 and Lamp2A (*60*, *61*). These proteins are also abundant in the membranes of late endosomes and MVBs (*62*, *63*). Therefore, it is conceivable that an analogous mechanism could act to import cytoplasmic Wnt7a into MVBs. However, endogenous Wnt7a proteins with an SP sequence are presumably trafficked through the ER and are secreted on EVs. This conundrum will require additional experiments to elucidate the precise mechanisms involved that traffic Wnt7a to EVs.

In conclusion, we have found an additional role for Coatomer components in the secretion of Wnts on EVs upon acute injury. We have elucidated the structural basis for the binding of Wnt7a to Coatomer and its secretion on the outer surface of EVs. Our results fill in a fundamental knowledge gap in the singularity of the long-range Wnt secretory pathway. We have defined the sequence requirements for Wnt-Coatomer interaction and shown that a similar mechanism is involved in EVs secretion of multiple Wnts. Moreover, our experiments suggest that the physiological secretion of Wnt7a in vivo during muscle regeneration is mediated by EVs, elucidating the ability of Wnts to signal over long distances in vivo.

Our experiments suggest that systemic delivery of Wnt7a loaded on EVs represents a potential therapy for treating neuromuscular diseases. The use of the EBP to direct the presentation of cargo proteins on the surface of EVs opens the door for multiple therapeutic applications. We anticipate that our discovery will be a starting point for more sophisticated delivery systems and providing important insight toward understanding Wnt secretion in pathological contexts.

## MATERIAL AND METHODS

### Mice

Experimental protocols OHRIe-3868 and OHRIb-3826 for mice used in this study were approved by the University of Ottawa Animal Care Committee in accordance with the guidelines of the Canadian Council on Animal Care. Food and water were administered ad libitum. Muscle regeneration experiments in Fig. 1 (A to C) were assessed in 8-week-old male C57BL/10ScSn M1 mice as previously described (*64*), with the following modifications. Mice were anesthetized with isoflurane, Cardiotoxin (CTX) injection was performed on a single injection into the TA (50 μl, 10 μM), and muscle regeneration was assessed after 96 hours. Muscle explant-derived EVs used in Fig. 1 (F to I) were obtained from F2 cross between the offspring of *Myf5-Cre* mice (*65*) and Wnt7a*^fl/fl^* mice (*26*) in a C57BL/6 genetic background. Briefly, mice were anesthetized with isoflurane, and CTX injection was performed on three injections (50 μl, 10 μM each), into the TA and one on each lobule of the gastrocnemius. Hindlimb muscles were harvested after 96 hours. In vivo assays in Fig. 6 (D to I) were performed in 12-week-old male C57BL/10ScSn-Dmdmdx/J mice from the Jackson Laboratory, more details in the “Electroporation of TA muscles” section.

### Cell cultures

HEK293T cells were obtained from American Type Culture Collection (ATCC) (CRL-3216) and verified to be free from mycoplasma contamination using the MycoSensor PCR Assay Kit (Agilent Technologies). Cells were cultured as in Dulbecco’s modified Eagle’s medium (DMEM) (Lonza) supplemented with 10% fetal bovine serum (FBS), penicillin (100 U/ml), and streptomycin (100 U/ml) and maintained at 37°C in a humidified incubator equilibrated with 5% CO_2_. Wild-type (WT) primary myoblasts were purified from C57BL/10ScSn M1 male mice by magnetic cell separation as previously described by Sincennes *et al.* (*66*). Primary myoblasts were cultured on collagen-coated dishes with HAM F12-X, 10% FBS, penicillin (100 U/ml), and streptomycin (100 U/ml) and maintained at 37°C in a humidified incubator equilibrated with 5% CO_2_. For differentiation, myoblasts were grown up to 80% confluence, and growth medium was replaced with differentiation medium [HAM F12-X/DMEM (1:1), 5% horse serum (HS), penicillin (100 U/ml), and streptomycin (100 U/ml)] for 4 days unless otherwise stated. During differentiation, serums were treated to be free of EVs before assays (*67*). Wnt7a-expressing primary myoblasts were obtained upon infection of primary myoblasts from C57BL/10ScSn M1 male mice, previously isolated as Sincennes *et al.* (*66*) described. Briefly, myoblasts passage 4 were seeded in six-well plate (1.0 × 10^5^ cells per well/4-ml media) and infected with lenti-III-Ubc-Wnt7a-HA lentivirus (30 μl virus per well) created in our laboratory and empty vector lentivirus as control containing polybrene (6 mg/ml) in 1.5-ml culture media [collagen-coated dishes with HAM F12-X, 10% FBS, penicillin (100 U/ml), streptomycin (100 U/mL) and maintained at 37°C in a humidified incubator equilibrated with 5% CO_2_]. Selection was done with puromycin (2.5 mg/ml) to establish the stable cell line. Six different colonies were picked, and Wnt7a expression was verified by immunoblot.

RPTEC-hTERT1 cells were culture in DMEM/F12 medium from ATCC plus the hTERT RPTEC Growth Kit from ATCC, G418 for immortalization selection, penicillin (100 U/ml), and streptomycin (100 U/ml) and maintained at 37°C in a humidified incubator equilibrated with 5% CO_2_.

### Pre-embedding immunogold labeling for tissue TEM

TA specimens were fixed in Karnovsky’s fixative for 2 weeks. After fixation, all segments were subsequently washed with 0.1 M sodium cacodylate and treated with 0.1% sodium borohydride in phosphate-buffered saline (PBS). Samples were permeabilized with 0.1% Triton X-100 and blocked with 10% donkey serum + 0.6% fish gelatin. TA samples were incubated with Wnt7a antibody. After a 48-hour incubation, segments were rinsed thoroughly with PBS and incubated overnight with the secondary antibody ultra-small (0.8 nm) gold conjugated (EMS) in blocking buffer at room temperature (RT). Later, samples were rinsed with 0.1 M sodium cacodylate and post-fixed with 2% glutaraldehyde in 0.1 M sodium cacodylate. Pre-embedding enhancement was realized with a silver enhancement kit (AURION R-Gent SE-EM, EMS) according to the manufacturer’s instructions. After enhancement, samples were secondly post-fixed with 1% osmium tetroxide in 0.1 M sodium cacodylate buffer. Then, samples were dehydrated in increasing concentration of ethanol and infiltrated in Spurr resin. Ultrathin transversal sections (80 nm) were collected onto 200-mesh copper grids and counterstained with 2% aqueous uranyl acetate and with Reynold’s lead citrate. Last, specimens were observed under a transmission electron microscope (Hitachi 7100, Gatan digital camera). For our analysis, approximately 50 immunoelectron micrographs were examined per muscle at different magnifications.

### Pre-embedding immunogold labeling for cells and EVs TEM

Fixed HEK293T cells/EVs pellets were treated separately with 0.1% sodium borohydride in PBS. Half of the specimen pellets were permeabilized with 0.1% Triton X-100 for 10 min, and the other half of the samples were processed without permeabilization. Pellets were blocked in a blocking buffer (10% donkey serum + 0.6% gelatin from cold water fish skin in PBS) for 2 hours. Pellets were incubated with the primary antibody for 48 hours. Pellets were incubated overnight with the secondary antibody (Jackson ImmunoResearch). Immunogold-labeled cells and EVs were fixed with 2% glutaraldehyde in 0.1 M sodium cacodylate buffer, and enhancement was performed with a silver enhancement kit on the immunogold-labeled cells. All samples were post-fixed with 1% osmium tetroxide in 0.1 M sodium cacodylate buffer. Specimens were dehydrated and embedded in resin and polymerized overnight at 70°C. Immunogold-labeled ultrathin sections of EVs were observed by transmission electron microscopy at 100,000× and 150,000×.

### Conditioned media production for cell-derived EVs

Equal numbers of HEK293T cells were seeded, and the different plasmids were transfected with linear polyethylenimine (Polysciences), accordingly to the manufacturer’s instructions. Later, transfected cells were cultured with 10% FBS EV-depleted (*67*) in DMEM (Gibco) and maintained at 37°C in a humidified incubator equilibrated with 5% CO_2_. After 48 hours of secretion, conditioned medium was collected for EVs isolation. For EVs derived from RPTEC-hTERT1 cells, equal numbers of cells were seeded and let them grow in growth media, as previously described. Upon 80% confluency, growth medium was change and maintained at 37°C in a humidified incubator equilibrated with 5% CO_2_ for 48 hours of secretion conditioned media. Then, medium was collected for EVs isolation following regular protocol.

### Production and isolation of EVs

For tissue EVs, we have standardized a protocol to obtain conditioned media from muscles explants (*25*). Briefly, 4 days after injury, both hindlimbs were harvested and cultured as explants on an EV-depleted FBS precoated dish with high-glucose DMEM (Gibco) and maintained at 37°C in a humidified incubator equilibrated with 5% CO_2_. After 48 hours, conditioned medium was collected for exosomal isolation.

Conditioned medium (20 ml) was clarified by sequential centrifugation (300*g* at RT for 10 min; 2500*g* at RT for 10 min; and 20,000*g* at 4°C for 20 min). Supernatant was transferred to Flexboy bag (Sartorius) and subjected to TFF under sterile conditions. Briefly, a KrosFlo Research 2i TFF system (Spectrum Laboratories) coupled to a MidGee Hoop ultrafiltration hollow fiber cartridge (GE Healthcare) 500-kDa cut off was used. Transmembrane pressure was automatically adjusted at 3 psi and a shear rate at 3000 s^−1^. Sample was concentrated up to 10 ml and then subjected to continuous diafiltration. Last, sample was concentrated at 5 ml and recovered from the cartridge. Last, EVs were pelleted down after spinning on an ultrabench centrifuge for 30 min at 100,000*g* at 4°C.

### Immunoblot analysis and immunohistochemistry

Immunoblot analysis was performed as described previously (*68*) with the following modifications. The lysates from EVs were not clarified by centrifugation. The immunoblot transferring was performed onto polyvinylidene difluoride membranes. All antibodies and dilutions are provided in table S3.

TA muscle cryosections were rehydrated using PBS and then fixed with 2% paraformaldehyde (PFA) in PBS at RT. After washing with PBS, permeabilization with a solution of 0.1% Triton and 0.1 M glycine in PBS was applied for 10 min at RT. Mouse on mouse blocking reagent was used at a dilution of 1:40 in a blocking solution of 10% goat serum, 1% bovine serum albumin (BSA), and 0.1% Tween 20 in PBS for 1 hour at RT. Primary antibodies were incubated overnight. Nuclei were counterstained with 4′,6-diamidino-2-phenylindole (DAPI) before mounting in Permafluor. For analysis, Z-stack images of cryosections were acquired on an epifluorescence microscope equipped with a motorized stage (Zeiss Axio Observer Z1) with a step size of 0.2 μm to span the cell (25 slices in total), and images were deconvoluted using Zen Software (Zeiss). The 3D sum intensity *Z* projection was performed with ImageJ software. Laminin staining was analyzed for fiber counting and minimum Feret’s diameter using SMASH (*69*).

HEK293T-transfected cells were fixed 10 min with 4% PFA, 10-min perm (0.1 M glycine and 0.1% Triton X-100 in PBS), and 1-hour blocking (5% HS and 2% BSA in PBS) at 4°C and incubated overnight in primary antibody and 1 hour in secondary antibody. Hoechst was used for nuclei counterstain before mounting in Permafluor. Images were acquired using a Zeiss LSM900 with Airyscan and 63× oil objective.

### Myotube hypertrophy assay

For Fig. 1 (H and I) wild-type primary myoblasts from C57BL/10ScSn M1 mice were differentiated for 4 days along with EVs stimulation at 10 μg/ml (based on total protein quantification after lysis) or recombinant Wnt7a protein at 100 ng/ml. For Fig. 6 (B and C), Wnt7a primary myoblasts from C57BL/10ScSn M1 mice, previously obtained as aforementioned, were transfected twice (every 24 hours two consecutive days) with *COPA*, *COPB2*, and *COPA* + *COPB2* siRNA at 10 nM (final concentration) using Lipofectamine RNAiMax, accordingly to the manufacturer’s instructions. After 3 days of differentiation, myotubes from both experiments were fixed with 4% PFA. Permeabilization and blocking solution consisting of 0.3 M glycine, 1% BSA, and 0.1% Tween 20 in PBS were added for 90 min. Pan myosin heavy chain primary antibody was incubated overnight. Nuclei were counterstained with DAPI before mounting in Permafluor.

For fig. S6M, primary myoblasts from C57BL/10ScSn M1 mice were seeded at a confluency of 100,000 cell per well in six-well plates. After 24 hours, myoblasts were differentiated for 4 days. HAMf10 and EV-free horse serum was supplemented with DMEM-conditioned media, previously collected from transfected HEK293T cells with the different plasmid control, Wnt7a, and Wnt7a_ΔEBP*GSGS, respectively. On the fourth day of differentiation, myotubes were fixed, permeabilized, and stained with pan-myosin heavy chain primary antibody overnight at 4°C. They were then incubated with secondary anti-mouse antibody conjugated with Alexa 546 for 1 hour at RT and lastly stained with DAPI.

For analysis, Z-stack images of myotubes were acquired on an epifluorescence microscope equipped with a motorized stage (Zeiss Axio Observer Z1) with a step size of 0.2 μm to span the cell (25 slices in total), and images were deconvoluted using Zen Software (Zeiss). The 3D sum intensity *Z* projection was performed with ImageJ software. Ten blind images were acquired per sample. The 50 largest myotubes from each well were included in the analysis. Fiji software was used to analyze myotube diameter. Three different samples were measured per condition.

### Construction of Wnt7a mutants

All constructs were cloned in a pcDNA3 vector. Wnt7a was originated from a pcDNA3-hWnt7a-HA plasmid that was designed in our laboratory. Wnt10a and Wnt16 were originated from pcDNA-hWnt10a-V5 (Addgene 35939) or pcDNA-hWnt16-V5 (Addgene 35942) plasmid, respectively. Wnt10b and Wnt3a constructs used in this publication were a gift from M. Waterman, D. Virshup, and X. He from the plasmid kit (*70*) (Addgene kit #1000000022). HALO*EBP-HA and HALO*EBP constructs were originated from a pBSM13-Pax7HALO plasmid that was designed in our laboratory. Mutation and truncation were generated by overlap extension polymerase chain reaction (PCR) with specially designed primers. Bam HI and Eco RI restriction sites were included in primers. PCR products and pcDNA3-HA vector were digested with Bam HI and Eco RI and ligated with Takara ligase solution. All constructs were verified by sequencing. All primers and coding sequences sources are provided in table S4.

### In silico homology modeling of Wnt7a

The homology model of human Wnt7a was constructed through its sequence annealing over the resolved structure of Wnt3 protein [Protein Data Bank (PDB) (*71*) and 6AHY (*72*)] with FoldX (*27*) BuildModel command. The annealing of the sequence resulted in no energetic conflicts enlighting that the folding captured by the crystal represents a stable configuration of proteins within the Wnt family. GSGS linker length was chosen to replace EBP. To affect folding, we considered the distance criteria respect to the terminal residues of the EBP.

### In silico determination of the EBP region

The in silico determination of the EBP region was performed through the free energy measurement of folding of the Wnt7a model (Δ*G*_wt_) versus the free energy resulting of the truncation of windows of 15 amino acid (Δ*G*_truncated_) along the whole sequence. Those regions not contributing to the protein folding present a very negative variation energy (ΔΔ*G*_truncated_WT_ ≪ 0). The N-terminal region is not structured since the mapping of the Wnt folding domain [PFAM (*73*) PF00110] starts in position 41, and the C-terminal region presents also low energies being a folded region not in close contact with the rest of the protein. Besides the terminal regions, the only sequence window presenting very low energy was selected as EBP (afterward confirmed experimentally) because it is not important for the folding and is highly variable along the Wnt family, evincing that its sequence codifies for functional behavior.

### Modeling of the EBP-loop swapping

All the unstructured regions within the Wnt7a generated model that were surrounded by secondary structured regions were evaluated in terms of end-to-end distances and torsional angles to establish their ability to room the EBP region through a sequence swap. Using ModelX (*74*) fragment replacement, the EBP was inserted using as anchoring terminal amino acids GLU171 and ASN175. Energies of the replaced model were measured then with the FoldX force field, and no energetic conflicts or clashes where found, demonstrating that the sequence swapping was supported by the structure.

### HALO uptake assay

HEK293T cells were transfected with pcDNA3_HALO and pcDNA3_HALO-EBP plasmids that we generated from a Pax7-HALO plasmid (Epoch Life Science) using polyethylenimine (PEI) as aforementioned. EVs from transfected cells were isolated as previously described and added to fresh seeded HEK293T for 15 min. Afterward, stimulated cells were labeled with HaloTag Ligands for Super Resolution Microscopy Janelia 549 (Promega) according to the manufacturer’s instructions. Cells were then fixed in 2% PFA for 5 min and washed three times with PBS. Last, cells were analyzed by image cytometry in the Amnis ImageStream X platform to verify the location of the fluorescence within the cytoplasm. The fluorescence detected by the Amnis ImageStream was excited using 561-nm laser and detected by the 580-30 emission filter channel.

### Drug inhibition of PORCN

HEK293T cells were transfected with pcDNA3_Wnt7a-FL-HA using PEI as aforementioned. After 6 hours of transfection, cells were treated overnight with PORCN inhibitor diluted in dimethyl sulfoxide (DMSO) (AdooQ) at two different concentrations, 100 and 500 nM in fresh media. Next morning, the medium was changed for EV secretion media and let them secrete for 48 hours.

### Knockdown by siRNA transfection

siRNA transfections for Fig. 4 were performed on HEK293T cells at 16 hours after culture using Lipofectamine RNAiMAX (Life Technologies) according to the manufacturer’s instructions. siRNAs for *WLS*, *COPA*, and *COPB2* were purchased from Dharmacon and used at a final concentration of 10, 10, and 20 nM, respectively. The following day, cells were transfected with Wnt7a as aforementioned, and Wnt7a secretion on EVs was tested 48 hours later.

siRNA transfections for Fig. 6A were performed on Wnt7a primary myoblasts from C57BL/10ScSn M1 mice, previously obtained as aforementioned. They were transfected twice (every 24 hours for two consecutive days) with *COPA*, *COPB2*, and *COPA* + *COPB2* siRNA at 10 nM (final concentration) using Lipofectamine RNAiMax according to the manufacturer’s instructions.

### BioID interactome analysis

Stable primary myoblast cell lines expressing BioID2, BioID2-EBP, or Wnt7a-BioID2 fusion proteins were generated using the mycBioID2-pBABE-puro vector (Addgene Plasmid). Myoblasts were grown in 15-cm culture dishes at subconfluency and incubated with biotin (Sigma-Aldrich: dissolved in DMSO) at a final concentration of 50 μM for 18 hours. Plates were scraped in ice-cold PBS, spun at 20,817*g* for 5 min to concentrate cell pellet, and then resuspended in radioimmunoprecipitation assay (RIPA) lysis buffer containing protease inhibitor cocktail. Cells were incubated on ice for 30 min and then spun down at 20,817*g*, 4°C for 20 min. Supernatant was transferred to low retention Eppendorf tube, and the protein concentration was quantified using Bradford reagent and spectrometry. Magnetic streptavidin beads (New England Biolabs) were used to precipitate the biotinylated protein fraction. Streptavidin beads were washed twice in RIPA lysis buffer and subsequently added to protein lysates for overnight incubation at 4°C with rotation. The following day, beads were sequentially washed with RIPA buffer, 1 M KCl, 0.1 M Na_2_CO_3_, 2 M urea in 10 mM tris-HCl (pH 8), and a final RIPA buffer wash. Biotinylated proteins were then eluted from beads by boiling for 10 min in 25 μl of 6x Laemmli buffer containing 20 mM dithiothreitol (DTT) and 2 mM biotin. Supernatant was loaded into precast gradient gel (4 to 15% Mini-PROTEAN TGX Stain-Free Protein Gel) and run for 30 min at 100 V. Colloidal blue dye (Thermo Fisher Scientific) was applied for 3 hours and then rinsed in Mili-Q water while shaking overnight. The entire protein containing lane for each condition was then cut out and stored in 1% acetic acid.

### Proteomic analysis

Proteins were digested in-gel using trypsin (Promega) according to the method of Shevchenko (*75*). Peptide extracts were concentrated by Vacufuge (Eppendorf) and purified by ZipTip (Sigma-Millipore). Liquid chromatography tandem mass spectrometry was performed using a Dionex Ultimate 3000 RLSC nano HPLC (Thermo Fisher Scientific) and Orbitrap Fusion Lumos mass spectrometer (Thermo Fisher Scientific). MASCOT software version 2.6.2 (Matrix Science) was used to infer peptide and protein identities from the mass spectra. The observed spectra were matched against sequences from SwissProt (version 2020-01) and against an in-house database of common contaminants. The results were exported to Scaffold (Proteome Software) for further validation and viewing. Enrichment heatmap was generated by computing the log_2_ of the fold enrichment of each condition versus its control. Gene Ontology term enrichment analysis was performed over the “cellular component” branch using ClueGO plugin on Cytoscape software.

### Proximity ligation assay

Fixed cells (murine primary myotubes, RPTEC-hTERT, or transfected HEK293T cells) were permeabilized (0.1% Triton X-100, 0.1 M glycine, and PBS) for 10 min and blocked with Duolink Blocking Solution (Sigma-Aldrich) for 3 hours. Incubation with primary antibodies diluted in Duolink Blocking Solution (Sigma-Aldrich) was performed overnight at 4°C. PLA reactions were subsequently performed using Duolink PLA probes for goat-mouse and goat-rabbit and Duolink In Situ Detection Reagents Red (Sigma-Aldrich) following the manufacturer’s protocol. Myotubes were counterstained with GM130 to visualize the Golgi apparatus. After the final wash, cells were mounted with VECTASHIELD Antifade Mounting Medium with DAPI (Vector Laboratories). For analysis, Z-stack images of myotubes were acquired on an epifluorescence microscope equipped with a motorized stage (Zeiss Axio Observer Z1) with a step size of 0.2 μm to span the cell (25 slices in total), and images were deconvoluted using Zen Software (Zeiss). The 3D sum intensity *Z* projection was performed with ImageJ software.

### Immunoprecipitation

Wnt7a-HA was overexpressed in HEK293T cells using Lipofectamine 2000 (Life Technologies) according to the manufacturer’s instructions. Cells and EVs were isolated 2 days after transfection and lysed in immunoprecipitation lysis buffer [50 mM tris (pH 7.5), 150 mM NaCl, 2 mM MgCl_2_, 0.5 mM EDTA, 0.5% Triton X-100, and protease inhibitors] for 30 min on ice. Lysates from cells were cleared by centrifugation and were incubated with either HA (Benthyl) or COPB2 (Cusabio) antibodies (Dynabeads Protein G, Thermo Fisher Scientific) overnight at 4°C, accordingly to the manufacturer’s instructions. Beads were washed four times with lysis buffer and eluted with Laemmli buffer. Immunoprecipitates were resolved by SDS–polyacrylamide gel electrophoresis and analyzed by immunoblot with the indicated antibodies.

### Recombinant protein production

The DNA sequence encoding COPB2_1–304_ was custom-synthesized with codon optimization for expression in *Escherichia coli* (GenScript) and cloned into pET28-Sumo3 vector (EMBL, Heidelberg) to express it with an N-terminal cleavable 6xHis-Sumo3 tag.

Native protein was expressed in *E. coli* BL21(DE3) grown in Luria-Bertani broth at 37°C, and protein expression was induced at an optical density at 600 nm of 0.8 by the addition of 0.5 mM isopropyl-β-d-thiogalactopyranoside. Cells were harvested after 16 hours of growth at 18°C. All following purification steps were performed at 4°C. The concentration of all purified proteins was calculated using the theoretical extinction coefficient.

For the purification of COPB2_1–304_, cell pellets were lysed at 4°C using high-pressure homogenization at 25 kpsi (1 kpsi = 69 _ 103 MPa) (Constant System Ltd, UK) in lysis buffer [50 mM tris-HCl (pH:7.5), 500 mM NaCl, 1 mM DTT, and 10 mM imidazole] and supplemented with 0.1 mM phenylmethylsulfonyl fluoride (PMSF) and 1 mM benzamidine. After centrifugation at 50,000*g* for 45 min, the soluble fraction was incubated for 2 hours in batch with Ni^2+^-nitrilotriacetate (NTA) agarose resin (Macherey-Nagel). After extensive washing of the beads with lysis buffer, the protein was eluted with lysis buffer supplemented with 250 mM imidazol. The N-terminal 6xHis-Sumo3-tag was subjected to overnight Sentrin-specific protease 2 digestion via dialysis into 150 mM NaCl, 1 mM DTT, 10 mM imidazole, and 25 mM tris-HCl (pH 7.5). A second Ni^2+^-NTA chromatography was carried out to remove the cleaved tag and uncleaved protein. COPB2_1–304_ was subsequently purified by ion-exchange chromatography (HitrapQ, GE Healthcare), diluting the NaCl concentration to 30 mM with buffer A (25 mM tris-HCl 7.0 and 1 mM DTT) and using a gradient of 30 to 1000 mM NaCl followed by size exclusion chromatography (Superdex 75 16/60, GE Healthcare) in buffer B [25 mM tris-HCl (pH 7.0), 150 mM NaCl, and 0.5 mM tris(2-carboxyethyl) phosphine (TCEP)].

### Crystallization and data collection

COPB2_1–304_ at 10 mg/ml (0.28 mM) was incubated with the Wnt_252–257_ peptide (LKIKKP) at 3 mM during 1 hour in 150 mM NaCl and 25 mM tris (pH 7.5) before setting the crystallization experiments. Initial screening was performed with a Mosquito robot (TTP, Labtech) in 96-well MRC plates (Molecular Dimensions) at 18°C. A total of 576 different crystallization conditions from commercial screenings (Molecular Dimensions, Hampton Research and QIAGEN) were tested. The sitting drop experiments were performed with 250 nl of protein/peptide plus 250 nl from the reservoir solution. Crystal hits were manually optimized with drops containing 2 μl of protein and 2 μl of reservoir solution. Best crystals were obtained in 0.1 M Hepes (pH 7.0) and 2.0 M AmSO_4_. Crystals were cryoprotected with 20% glycerol, and diffraction data were collected in the XALOC beamline at ALBA synchrotron (table S2). Diffraction data were indexed, integrated, and scaled in P1 using XDS (*45*). PHASER (*76*) was used to solve the structure by molecular replacement using COPB2_1–304_ (PDB 2YNN) as a search model. The final model was obtained after several cycles of manual modification using COOT (*77*), followed by refinement using six twin operators in REFMAC (*78*). The coordinates and structure factors have been deposited at the PDB with the entry code PDB: 8R8B.

### ITC assays

ITC experiments were carried out on a VP-ITC titration microcalorimeter (MicroCal/GE Healthcare) at 25°C. COPB2_1–304_ and peptides used for ITC experiments were dialyzed overnight at 4°C against 150 mM NaC, 0.5 mM TCEP, and 25 mM Hepes (pH 7.5) and degassed for 5 min in a ThermoVac sample degasser before titration. The titration sequence consisted of an initial 2-μl injection to prevent artifacts arising from filling of the syringe (not used in data fitting), followed by 28 injections of 10-μl aliquots with a spacing of 210 s between injections. Similar injections of protein or peptides in buffer were performed to determine the heat of dilution used to correct the experimental data. The resulting titration data were integrated and fitted to a one-site model using the Origin ITC software package supplied by MicroCal. The binding constant [acid constant (*K*_a_), *K*_d_ = 1/*K*_a_], the molar binding stoichiometry (*n*) and binding enthalpy (Δ*H*) were extracted directly from the fit. The free energy (Δ*G*) and entropy (Δ*S*) of binding were calculated from Δ*G* = −*RT*lnKa = Δ*H*-*T*Δ*S*, where *R* is the gas constant and *T* is the absolute temperature. The interaction of Wnt peptides with COPB2_1–304_ was analyzed by titrating 450 μM of each peptide into 20 μM COPB2_1–304_. Data are the mean of a minimum of three replicate titrations for each experiment.

### Electroporation of TA muscles

Right TA muscle was electroporated either with 30 μg of pcDNA3_Wnt7a or pcDNA3_ Wnt7a_ΔEBP*GSGS. Contralateral legs were electroporated with empty vector pcDNA3. All plasmids were purified using endotoxin-free kits (QIAGEN) and diluted in saline. A total of 50 μl of plasmid was injected intramuscularly into the TA of *mdx* mice under general anesthesia. Electric stimulation was applied with a pulse generator (ECM 830, BTX) of 100 to 150 V for six pulses, with a fixed duration of 20 ms and an interval of 200 ms using 5-mm needle electrodes (BTX).

### Statistical analysis

Experiments were performed with a minimum of three biological replicates, and results are presented as the means ± SEM. Student’s *t* test were performed to assess the statistical significance of two-tailed analysis. For multiple comparisons, two-way analysis of variance (ANOVA) test was used, and for post hoc analysis, Šidák’s multiple comparisons test was used. *P* values are indicated as *P ≤ 0.05, **P ≤ 0.01, and ***P ≤ 0.001, and *P* values <0.05 were considered statistically significant.

## References

[1] M. D. Wellenstein, S. B. Coffelt, D. E. M. Duits, M. H. van Miltenburg, M. Slagter, I. de Rink, L. Henneman, S. M. Kas, S. Prekovic, C.-S. Hau, K. Vrijland, A. P. Drenth, R. de Korte-Grimmerink, E. Schut, I. van der Heijden, W. Zwart, L. F. A. Wessels, T. N. Schumacher, J. Jonkers, K. E. de Visser, Loss of p53 triggers WNT-dependent systemic inflammation to drive breast cancer metastasis. Nature 572, 538–542 (2019). 31367040 10.1038/s41586-019-1450-6 PMC6707815

[2] K. Willert, R. Nusse, Wnt proteins. Cold Spring Harb. Perspect. Biol. 4, a007864 (2012). 22952392 10.1101/cshperspect.a007864 PMC3428774

[3] A. Polesskaya, P. Seale, M. A. Rudnicki, Wnt signaling induces the myogenic specification of resident CD45+ adult stem cells during muscle regeneration. Cell 113, 841–852 (2003). 12837243 10.1016/s0092-8674(03)00437-9

[4] C. Y. Logan, R. Nusse, The Wnt signaling pathway in development and disease. Annu. Rev. Cell Dev. Biol. 20, 781–810 (2004). 15473860 10.1146/annurev.cellbio.20.010403.113126

[5] A. H. Nile, R. N. Hannoush, Fatty acylation of Wnt proteins. Nat. Chem. Biol. 12, 60–69 (2016). 26784846 10.1038/nchembio.2005

[6] D. Routledge, S. Scholpp, Mechanisms of intercellular wnt transport. Development 146, dev176073 (2019). 31092504 10.1242/dev.176073

[7] K. Kaiser, D. Gyllborg, J. Procházka, A. Sala, P. Kompaníková, F. L. Molina, R. Laguna-goya, T. Radaszkiewicz, J. Harno, M. Procházková, D. Pot, R. A. Barker, Á. G. Casado, R. Sedlá, E. Arenas, J. C. Villaescusa, WNT5A is transported via lipoprotein particles in the cerebrospinal fl uid to regulate hindbrain morphogenesis. 1498 (2019). 10.1038/s41467-019-09298-4 PMC6445127 30940800

[8] D. Panáková, H. Sprong, E. Marois, C. Thiele, S. Eaton, Lipoprotein particles are required for Hedgehog and Wingless signalling. Nature 435, 58–65 (2005). 15875013 10.1038/nature03504

[9] B. Mattes, Y. Dang, G. Greicius, L. T. Kaufmann, B. Prunsche, J. Rosenbauer, J. Stegmaier, R. Mikut, S. Özbek, G. U. Nienhaus, A. Schug, D. M. Virshup, S. Scholpp, Wnt/PCP controls spreading of Wnt/β-catenin signals by cytonemes in vertebrates. eLife 7, e36953–e36928 (2018). 30060804 10.7554/eLife.36953 PMC6086664

[10] I. J. Mcgough, L. Vecchia, B. Bishop, T. Malinauskas, K. Beckett, D. Joshi, N. O’Reilly, C. Siebold, E. Y. Jones, J.-P. Vincent, Glypicans shield the Wnt lipid moiety to enable signalling at a distance. Nature 585, 85–90 (2020). 32699409 10.1038/s41586-020-2498-z PMC7610841

[11] K. A. Mulligan, C. Fuerer, W. Ching, M. Fish, K. Willert, R. Nusse, Secreted Wingless-interacting molecule (Swim) promotes long-range signaling by maintaining Wingless solubility. Proc. Natl. Acad. Sci. U.S.A. 109, 370–377 (2012). 22203956 10.1073/pnas.1119197109 PMC3258625

[12] J. C. Gross, V. Chaudhary, K. Bartscherer, M. Boutros, Active Wnt proteins are secreted on exosomes. Nat. Cell Biol. 14, 1036–1045 (2012). 22983114 10.1038/ncb2574

[13] V. Luga, L. Zhang, A. M. Viloria-Petit, A. A. Ogunjimi, M. R. Inanlou, E. Chiu, M. Buchanan, A. N. Hosein, M. Basik, J. L. Wrana, Exosomes mediate stromal mobilization of autocrine Wnt-PCP signaling in breast cancer cell migration. Cell 151, 1542–1556 (2012). 23260141 10.1016/j.cell.2012.11.024

[14] K. Menck, F. Klemm, J. C. Gross, T. Pukrop, D. Wenzel, C. Binder, Induction and transport of Wnt 5a during macrophage-induced malignant invasion is mediated by two types of extracellular vesicles. Oncotarget 4, 2057–2066 (2013). 24185202 10.18632/oncotarget.1336 PMC3875769

[15] G. Van Niel, G. D’Angelo, G. Raposo, Shedding light on the cell biology of extracellular vesicles. Nat. Rev. Mol. Cell Biol. 19, 213–228 (2018). 29339798 10.1038/nrm.2017.125

[16] A. M. Pani, B. Goldstein, Direct visualization of a native Wnt in vivo reveals that a long-range Wnt gradient forms by extracellular dispersal. eLife 7, e38325 (2018). 30106379 10.7554/eLife.38325 PMC6143344

[17] K. Beckett, S. Monier, L. Palmer, C. Alexandre, H. Green, G. Raposo, P. Thibault, R. Le Borgne, J. Vincent, Europe PMC Funders Group Drosophila Wingless is loaded on exosome-like vesicles but forms a gradient in an exosome-independent manner. 14, 82–96 (2015). 10.1111/tra.12016 PMC4337976 23035643

[18] C. Korkut, B. Ataman, P. Ramachandran, J. Ashley, R. Barria, N. Gherbesi, V. Budnik, *Trans*-synaptic transmission of vesicular Wnt signals through Evi/Wntless. Cell 139, 393–404 (2009). 19837038 10.1016/j.cell.2009.07.051 PMC2785045

[19] N. C. Chang, F. P. Chevalier, M. A. Rudnicki, Satellite cells in muscular dystrophy–Lost in polarity. Trends Mol. Med. 22, 479–496 (2016). 27161598 10.1016/j.molmed.2016.04.002 PMC4885782

[20] C. F. Bentzinger, J. von Maltzahn, N. A. Dumont, D. A. Stark, Y. X. Wang, K. Nhan, J. Frenette, D. D. W. Cornelison, M. A. Rudnicki, Wnt7a stimulates myogenic stem cell motility and engraftment resulting in improved muscle strength. J. Cell Biol. 205, 97–111 (2014). 24711502 10.1083/jcb.201310035 PMC3987134

[21] J. von Maltzahn, C. F. Bentzinger, M. A. Rudnicki, Wnt7a-Fzd7 signalling directly activates the Akt/mTOR anabolic growth pathway in skeletal muscle. Nat. Cell Biol. 14, 186–191 (2012). 10.1038/ncb2404 PMC3271181 22179044

[22] J. von Maltzahn, J.-M. Renaud, G. Parise, M. A. Rudnicki, Wnt7a treatment ameliorates muscular dystrophy. Proc. Natl. Acad. Sci. U.S.A. 109, 20614–20619 (2012). 23185011 10.1073/pnas.1215765109 PMC3528612

[23] C. Y. Janda, D. Waghray, A. M. Levin, C. Thomas, K. C. Garcia, Structural basis of Wnt recognition by frizzled. Science 337, 59–64 (2012). 22653731 10.1126/science.1222879 PMC3577348

[24] F. Le Grand, A. E. Jones, V. Seale, A. Scimè, M. A. Rudnicki, Wnt7a activates the planar cell polarity pathway to drive the symmetric expansion of satellite stem cells. Cell Stem Cell 4, 535–547 (2009). 19497282 10.1016/j.stem.2009.03.013 PMC2743383

[25] U. Gurriaran-Rodriguez, Y. De Repentigny, R. Kothary, M. A. Rudnicki, Isolation of small extracellular vesicles from regenerating muscle tissue using tangential flow filtration and size exclusion chromatography. *Skelet. Muscle* **14**, 22 (2024). 10.1186/s13395-024-00355-1 PMC11468478 39394606

[26] K. A. Dunlap, J. Filant, K. Hayashi, E. B. Rucker III, G. Song, J. M. Deng, R. R. Behringer, F. J. DeMayo, J. Lydon, J.-W. Jeong, T. E. Spencer, Postnatal deletion of *Wnt7a* inhibits uterine gland morphogenesis and compromises adult fertility in mice. Biol. Reprod. 85, 386–396 (2011). 21508348 10.1095/biolreprod.111.091769 PMC3142262

[27] J. Delgado, L. G. Radusky, D. Cianferoni, L. Serrano, FoldX 5.0: Working with RNA, small molecules and a new graphical interface. Bioinformatics 35, 4168–4169 (2019). 30874800 10.1093/bioinformatics/btz184 PMC6792092

[28] G. V. Los, L. P. Encell, M. G. McDougall, D. D. Hartzell, N. Karassina, C. Zimprich, M. G. Wood, R. Learish, R. F. Ohana, M. Urh, D. Simpson, J. Mendez, K. Zimmerman, P. Otto, G. Vidugiris, J. Zhu, A. Darzins, D. H. Klaubert, R. F. Bulleit, K. V. Wood, HaloTag: A novel protein labeling technology for cell imaging and protein analysis. ACS Chem. Biol. 3, 373–382 (2008). 18533659 10.1021/cb800025k

[29] K. Willert, J. D. Brown, E. Danenberg, A. W. Duncan, I. L. Weissman, T. Reya, J. R. Yates III, R. Nusse, Wnt proteins are lipid-modi ed and can act as stem cell growth factors. Nature 423, 448–452 (2003). 12717451 10.1038/nature01611

[30] H. Komekado, H. Yamamoto, T. Chiba, A. Kikuchi, Glycosylation and palmitoylation of Wnt-3a are coupled to produce an active form of Wnt-3a. Genes Cells 12, 521–534 (2007). 17397399 10.1111/j.1365-2443.2007.01068.x

[31] R. Takada, Y. Satomi, T. Kurata, N. Ueno, S. Norioka, H. Kondoh, T. Takao, S. Takada, Monounsaturated fatty acid modification of Wnt protein: Its role in Wnt secretion. 11, 791–801 (2006). 10.1016/j.devcel.2006.10.003 17141155

[32] J. von Maltzahn, R. Zinoviev, N. C. Chang, C. F. Bentzinger, M. A. Rudnicki, A truncated Wnt7a retains full biological activity in skeletal muscle. Nat. Commun. 4, 2869 (2013). 24287629 10.1038/ncomms3869 PMC3868162

[33] J. Liu, S. Pan, M. H. Hsieh, N. Ng, F. Sun, T. Wang, S. Kasibhatla, A. G. Schuller, A. G. Li, D. Cheng, J. Li, C. Tompkins, A. Pferdekamper, A. Steffy, J. Cheng, C. Kowal, V. Phung, G. Guo, Y. Wang, M. P. Graham, S. Flynn, J. C. Brenner, C. Li, M. C. Villarroel, P. G. Schultz, X. Wu, P. McNamara, W. R. Sellers, L. Petruzzelli, A. L. Boral, H. M. Seidel, M. E. McLaughlin, J. Che, T. E. Carey, G. Vanasse, J. L. Harris, Targeting Wnt-driven cancer through the inhibition of Porcupine by LGK974. Proc. Natl. Acad. Sci. U.S.A. 110, 20224–20229 (2013). 24277854 10.1073/pnas.1314239110 PMC3864356

[34] K. Bartscherer, N. Pelte, D. Ingelfinger, M. Boutros, Secretion of Wnt ligands requires evi, a conserved transmembrane protein. Cell 125, 523–533 (2006). 16678096 10.1016/j.cell.2006.04.009

[35] C. Bänziger, D. Soldini, C. Schütt, P. Zipperlen, G. Hausmann, K. Basler, Wntless, a conserved membrane protein dedicated to the secretion of Wnt proteins from signaling cells. Cell 125, 509–522 (2006). 16678095 10.1016/j.cell.2006.02.049

[36] R. Nygaard, J. Yu, J. Kim, D. Ross, G. Parisi, O. B. Clarke, D. M. Virshup, F. Mancia, Structural basis of WLS/evi-mediated Wnt transport and secretion. Cell 184, 194–206.e14 (2021). 33357447 10.1016/j.cell.2020.11.038 PMC7797000

[37] M. Mathieu, N. Névo, M. Jouve, J. I. Valenzuela, M. Maurin, F. J. Verweij, R. Palmulli, D. Lankar, F. Dingli, D. Loew, E. Rubinstein, G. Boncompain, F. Perez, C. Théry, Specificities of exosome versus small ectosome secretion revealed by live intracellular tracking of CD63 and CD9. Nat. Commun. 12, 4389 (2021). 34282141 10.1038/s41467-021-24384-2 PMC8289845

[38] T. C. Branon, J. A. Bosch, A. D. Sanchez, N. D. Udeshi, T. Svinkina, S. A. Carr, J. L. Feldman, N. Perrimon, A. Y. Ting, Efficient proximity labeling in living cells and organisms with TurboID. Nat. Biotechnol. 36, 880–887 (2018). 30125270 10.1038/nbt.4201 PMC6126969

[39] S. Chun, S. Ahn, C.-H. Yeom, S. Park, Exosome proteome of U-87MG glioblastoma cells. Biology 5, 50 (2016). 27929413 10.3390/biology5040050 PMC5192430

[40] B. J. Tauro, D. W. Greening, R. A. Mathias, S. Mathivanan, H. Ji, R. J. Simpson, Two distinct populations of exosomes are released from LIM1863 colon carcinoma cell-derived organoids. Mol. Cell. Proteomics 12, 587–598 (2013). 23230278 10.1074/mcp.M112.021303 PMC3591653

[41] F. Gu, J. Gruenberg, ARF1 regulates pH-dependent COP functions in the early endocytic pathway. J. Biol. Chem. 275, 8154–8160 (2000). 10713138 10.1074/jbc.275.11.8154

[42] J. Aniento, F. Gu, R. G. Parton, J. Gruenberg, An endosomal beta COP is involved in the pH-dependent formation of transport vesicles destined for late endosomes. J. Cell Biol. 133, 29–41 (1996). 8601610 10.1083/jcb.133.1.29 PMC2120778

[43] F. Gu, F. Aniento, R. G. Parton, J. Gruenberg, Functional dissection of COP-I subunits in the biogenesis of multivesicular endosomes. J. Cell Biol. 139, 1183–1195 (1997). 9382865 10.1083/jcb.139.5.1183 PMC2140201

[44] W. Ma, J. Goldberg, Rules for the recognition of dilysine retrieval motifs by coatomer. EMBO J. 32, 926–937 (2013). 23481256 10.1038/emboj.2013.41 PMC3616288

[45] W. Kabsch, XDS. Acta Crystallogr. D Biol. Crystallogr. 66, 125–132 (2010). 20124692 10.1107/S0907444909047337 PMC2815665

[46] E. S. Witze, E. S. Litman, G. M. Argast, R. T. Moon, N. G. Ahn, Wnt5a control of cell polarity and directional movement by polarized redistribution of adhesion receptors. Science 320, 365–369 (2008). 18420933 10.1126/science.1151250 PMC3229220

[47] E. S. Witze, M. K. Connacher, S. Houel, M. P. Schwartz, M. K. Morphew, L. Reid, D. B. Sacks, K. S. Anseth, N. G. Ahn, Wnt5a directs polarized calcium gradients by recruiting cortical endoplasmic reticulum to the cell trailing edge. Dev. Cell 26, 645–657 (2013). 24091015 10.1016/j.devcel.2013.08.019 PMC3884950

[48] H.-L. Chen, J.-J. Li, F. Jiang, W.-J. Shi, G.-Y. Chang, MicroRNA-4461 derived from bone marrow mesenchymal stem cell exosomes inhibits tumorigenesis by downregulating COPB2 expression in colorectal cancer. Biosci. Biotechnol. Biochem. 84, 338–346 (2020). 31631786 10.1080/09168451.2019.1677452

[49] J. Zhang, J.-P. Andersen, H. Sun, X. Liu, N. Sonenberg, J. Nie, Y. Shi, Aster-C coordinates with COP I vesicles to regulate lysosomal trafficking and activation of mTORC1. EMBO Rep. 21, e49898 (2020). 32648345 10.15252/embr.201949898 PMC7507422

[50] M. Eubelen, N. Bostaille, P. Cabochette, A. Gauquier, P. Tebabi, A. C. Dumitru, M. Koehler, P. Gut, D. Alsteens, D. Y. R. Stainier, A. Garcia-Pino, B. Vanhollebeke, A molecular mechanism for Wnt ligand-specific signaling. Science 361, eaat1178 (2018). 30026314 10.1126/science.aat1178

[51] M. Vallon, K. Yuki, T. D. Nguyen, J. Chang, J. Yuan, D. Siepe, Y. Miao, M. Essler, M. Noda, K. C. Garcia, C. J. Kuo, A RECK-WNT7 receptor-ligand interaction enables isoform-specific regulation of Wnt bioavailability. Cell Rep. 25, 339–349.e9 (2018). 30304675 10.1016/j.celrep.2018.09.045 PMC6338448

[52] M. L.-H. Chu, V. E. Ahn, H.-J. Choi, D. L. Daniels, R. Nusse, W. I. Weis, Structural studies of wnts and identification of an LRP6 binding site. Structure 21, 1235–1242 (2013). 23791946 10.1016/j.str.2013.05.006 PMC3731992

[53] C. F. Bentzinger, Y. X. Wang, J. von Maltzahn, V. D. Soleimani, H. Yin, M. A. Rudnicki, Fibronectin regulates Wnt7a signaling and satellite cell expansion. Cell Stem Cell 12, 75–87 (2013). 23290138 10.1016/j.stem.2012.09.015 PMC3539137

[54] P. E. Wright, H. J. Dyson, Intrinsically disordered proteins in cellular signalling and regulation. Nat. Rev. Mol. Cell Biol. 16, 18–29 (2015). 25531225 10.1038/nrm3920 PMC4405151

[55] J. Yu, J. Chia, C. A. Canning, C. M. Jones, F. A. Bard, D. M. Virshup, WLS retrograde transport to the endoplasmic reticulum during Wnt secretion. Dev. Cell 29, 277–291 (2014). 24768165 10.1016/j.devcel.2014.03.016

[56] M. Schmidt, C. Poser, C. Janster, J. von Maltzahn, The hairpin region of WNT7A is sufficient for binding to the Frizzled7 receptor and to elicit signaling in myogenic cells. Comput. Struct. Biotechnol. J. 20, 6348–6359 (2022). 36420144 10.1016/j.csbj.2022.10.047 PMC9678774

[57] W. Ching, H. C. Hang, R. Nusse, Lipid-independent secretion of a *Drosophila* Wnt protein. J. Biol. Chem. 283, 17092–17098 (2008). 18430724 10.1074/jbc.M802059200 PMC2427328

[58] M. H. Richards, M. S. Seaton, J. Wallace, L. Al-Harthi, Porcupine is not required for the production of the majority of wnts from primary human astrocytes and CD8+ T cells. PLOS ONE 9, e92159 (2014). 24647048 10.1371/journal.pone.0092159 PMC3960167

[59] H. Yao, J. Lan, C. Li, H. Shi, J.-P. Brosseau, H. Wang, H. Lu, C. Fang, Y. Zhang, L. Liang, X. Zhou, C. Wang, Y. Xue, Y. Cui, J. Xu, Inhibiting PD-L1 palmitoylation enhances T-cell immune responses against tumours. Nat. Biomed. Eng. 3, 306–317 (2019). 30952982 10.1038/s41551-019-0375-6

[60] L. Qiao, J. Hu, X. Qiu, C. Wang, J. Peng, C. Zhang, M. Zhang, H. Lu, W. Chen, LAMP2A, LAMP2B and LAMP2C: Similar structures, divergent roles. Autophagy 19, 2837–2852 (2023). 37469132 10.1080/15548627.2023.2235196 PMC10549195

[61] N. Salvador, C. Aguado, M. Horst, E. Knecht, Import of a cytosolic protein into lysosomes by chaperone-mediated autophagy depends on its folding state. J. Biol. Chem. 275, 27447–27456 (2000). 10862611 10.1074/jbc.M001394200

[62] J. V. Ferreira, A. da Rosa Soares, J. Ramalho, C. Máximo Carvalho, M. H. Cardoso, P. Pintado, A. S. Carvalho, H. C. Beck, R. Matthiesen, M. Zuzarte, H. Girão, G. van Niel, P. Pereira, LAMP2A regulates the loading of proteins into exosomes. Sci. Adv. 8, eabm1140 (2022). 35333565 10.1126/sciadv.abm1140 PMC8956266

[63] R. Sahu, S. Kaushik, C. C. Clement, E. S. Cannizzo, B. Scharf, A. Follenzi, I. Potolicchio, E. Nieves, A. M. Cuervo, L. Santambrogio, Microautophagy of cytosolic proteins by late endosomes. Dev. Cell 20, 131–139 (2011). 21238931 10.1016/j.devcel.2010.12.003 PMC3025279

[64] U. Gurriarán-Rodríguez, I. Santos-Zas, O. Al-Massadi, C. S. Mosteiro, D. Beiroa, R. Nogueiras, A. B. Crujeiras, L. M. Seoane, J. Señarís, T. García-Caballero, R. Gallego, F. F. Casanueva, Y. Pazos, J. P. Camiña, The obestatin/GPR39 system is up-regulated by muscle injury and functions as an autocrine regenerative system. J. Biol. Chem. 287, 38379–38389 (2012). 22992743 10.1074/jbc.M112.374926 PMC3488106

[65] S. Kuang, K. Kuroda, F. Le Grand, M. A. Rudnicki, Asymmetric self-renewal and commitment of satellite stem cells in muscle. Cell 129, 999–1010 (2007). 17540178 10.1016/j.cell.2007.03.044 PMC2718740

[66] M. C. Sincennes, Y. X. Wang, M. A. Rudnicki, Primary mouse myoblast purification using magnetic cell separation, in *Muscle Stem Cells*: *Methods and Protocols*, E. Perdiguero, D. D. W. Cornelison, Eds. (Springer, 2017), pp. 41–50. 10.1007/978-1-4939-6771-1_3 28247344

[67] C. Théry, S. Amigorena, G. Raposo, A. Clayton, Isolation and characterization of exosomes from cell culture supernatants. Curr. Protoc. Cell Biol., 10.1002/0471143030.cb0322s30, (2006). 18228490

[68] U. Gurriarán-Rodríguez, I. Santos-Zas, J. González-Sánchez, D. Beiroa, V. Moresi, C. S. Mosteiro, W. Lin, J. E. Viñuela, J. Señarís, T. García-Caballero, F. F. Casanueva, R. Nogueiras, R. Gallego, J.-M. Renaud, S. Adamo, Y. Pazos, J. P. Camiña, Action of obestatin in skeletal muscle repair: Stem cell expansion, muscle growth, and microenvironment remodeling. Mol. Ther. 23, 1003–1021 (2015). 25762009 10.1038/mt.2015.40 PMC4817756

[69] L. R. Smith, E. R. Barton, SMASH - semi-automatic muscle analysis using segmentation of histology: A MATLAB application. Skelet Muscle 4, 21 (2014). 25937889 10.1186/2044-5040-4-21 PMC4417508

[70] B. T. MacDonald, A. Hien, X. Zhang, O. Iranloye, D. M. Virshup, M. L. Waterman, X. He, Disulfide bond requirements for active Wnt ligands. J. Biol. Chem. 289, 18122–18136 (2014). 24841207 10.1074/jbc.M114.575027 PMC4140276

[71] H. Berman, K. Henrick, H. Nakamura, J. L. Markley, The worldwide protein data bank (wwPDB): Ensuring a single, uniform archive of PDB data. Nucleic Acids Res. 35, D301–D303 (2007). 17142228 10.1093/nar/gkl971 PMC1669775

[72] H. Hirai, K. Matoba, E. Mihara, T. Arimori, J. Takagi, Crystal structure of a mammalian Wnt–frizzled complex. Nat. Struct. Mol. Biol. 26, 372–379 (2019). 31036956 10.1038/s41594-019-0216-z

[73] A. Bateman, L. Coin, R. Durbin, R. D. Finn, V. Hollich, S. Griffiths-Jones, A. Khanna, M. Marshall, S. Moxon, E. L. L. Sonnhammer, D. J. Studholme, C. Yeats, S. R. Eddy, The Pfam protein families database. Nucleic Acids Res. 32, D138–D141 (2004). 14681378 10.1093/nar/gkh121 PMC308855

[74] J. D. Blanco, L. G. Radusky, D. Cianferoni, L. Serrano, Protein-assisted RNA fragment docking (RnaX) for modeling RNA–protein interactions using ModelX. Proc. Natl. Acad. Sci. U.S.A. 116, 24568–24573 (2019). 31732673 10.1073/pnas.1910999116 PMC6900601

[75] A. Shevchenko, H. Tomas, J. Havliš, J. V. Olsen, M. Mann, In-gel digestion for mass spectrometric characterization of proteins and proteomes. Nat. Protoc. 1, 2856–2860 (2006). 17406544 10.1038/nprot.2006.468

[76] P. D. Adams, P. V. Afonine, G. Bunkóczi, V. B. Chen, I. W. Davis, N. Echols, J. J. Headd, L.-W. Hung, G. J. Kapral, R. W. Grosse-Kunstleve, A. J. McCoy, N. W. Moriarty, R. Oeffner, R. J. Read, D. C. Richardson, J. S. Richardson, T. C. Terwilliger, P. H. Zwart, PHENIX: A comprehensive Python-based system for macromolecular structure solution. Acta Crystallogr. D Biol. Crystallogr. 66, 213–221 (2010). 20124702 10.1107/S0907444909052925 PMC2815670

[77] P. Emsley, B. Lohkamp, W. G. Scott, K. Cowtan, Features and development of coot. Acta Crystallogr. D Biol. Crystallogr. 66, 486–501 (2010). 20383002 10.1107/S0907444910007493 PMC2852313

[78] P. Skubák, G. N. Murshudov, N. S. Pannu, Direct incorporation of experimental phase information in model refinement. Acta Crystallogr. D Biol. Crystallogr. 60, 2196–2201 (2004). 15572772 10.1107/S0907444904019079

