# Supplementary material for: Identification of the Wnt signal peptide that directs secretion on extracellular vesicles
Source: Sci Adv. 2024 Dec 11;10(50):eado5914. doi: 10.1126/sciadv.ado5914 (PMC11633749; doi:10.1126/sciadv.ado5914)
Supplement: Supplementary file 1 — Figs. S1 to S6 Tables S1 to S4 [file sciadv.ado5914_sm.pdf]

Science Advances
AAAS

Supplementary Materials for

Identification of the Wnt signal peptide that directs secretion on extracellular vesicles

Uxia Gurriaran-Rodriguez *et al.*

Corresponding author: Michael A. Rudnicki, mrudnicki@ohri.ca

Sci. Adv. **10**, eado5914 (2024)
DOI: 10.1126/sciadv.ado5914

This PDF file includes:

Figs. S1 to S6
Tables S1 to S4

Supplemental Figure S1

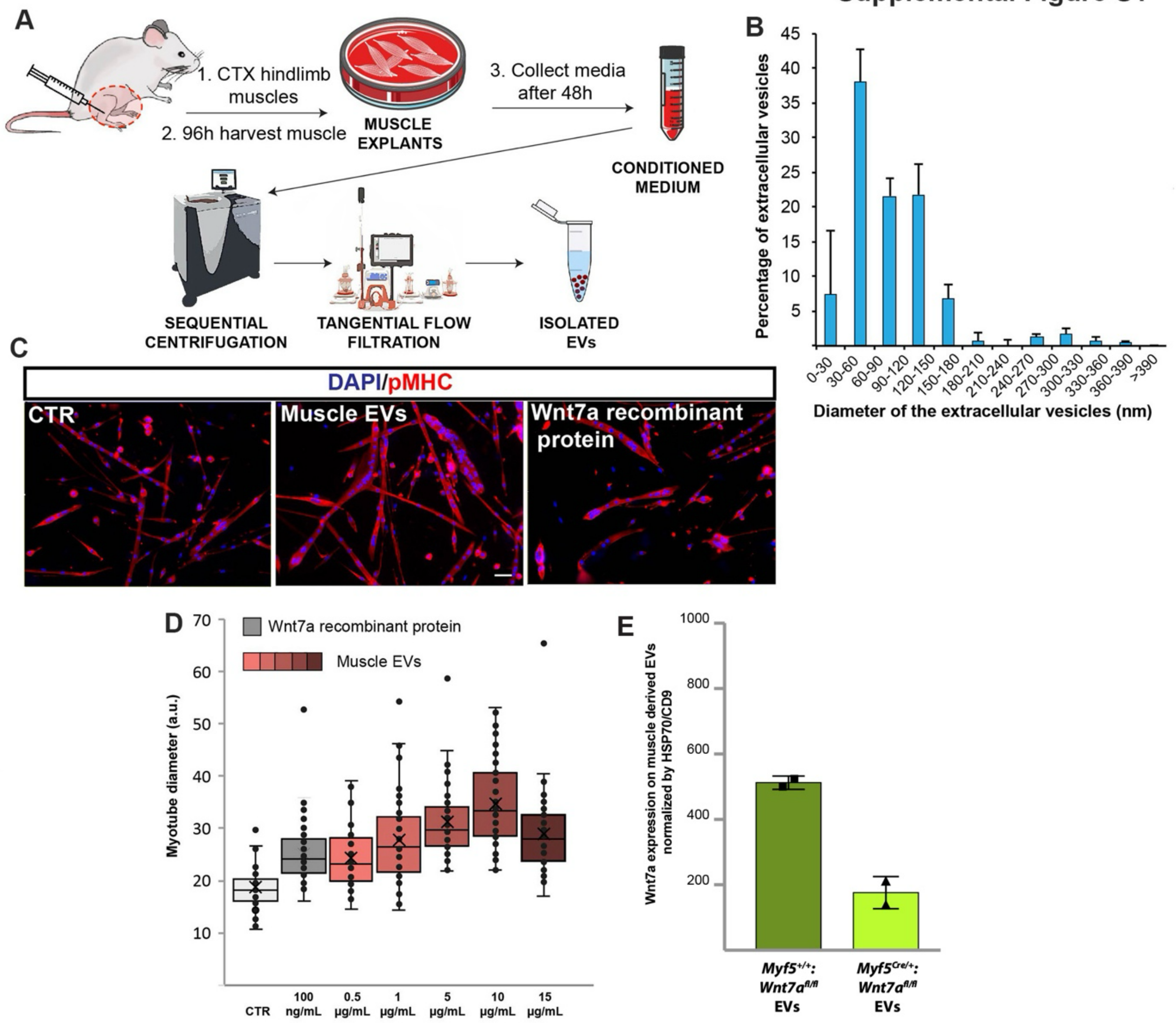

Fig. S1. Regenerating muscle secretes Wnt7a. (**A**) Experimental protocol used to obtain EVs from mice hind limb muscle. (**B**) Relative size distribution analysis of EVs fraction from muscle explants. n = 3 biological replicates (**C**) pMHC immunofluorescence representative images of hypertrophied myotubes after muscle EVs stimulation. Scale bar 50µm. (**D**) Hypertrophy dose-response assay of murine primary myotubes treated with muscle EVs. Data shown as fold change on myotube diameter over the control (%); Wnt7a recombinant protein was used as a positive control. n = 50 technical replicates **(E)** Quantification of Wnt7a expression abrogation in EVs isolated *from Myf5*$^{(Cre/+)}$*: Wnt7a*$^{(fl/fl)}$ hind limb muscle at 96 h post-CTX injury from Fig 1G. n = 2 mice. Data are mean ± s.e.m.

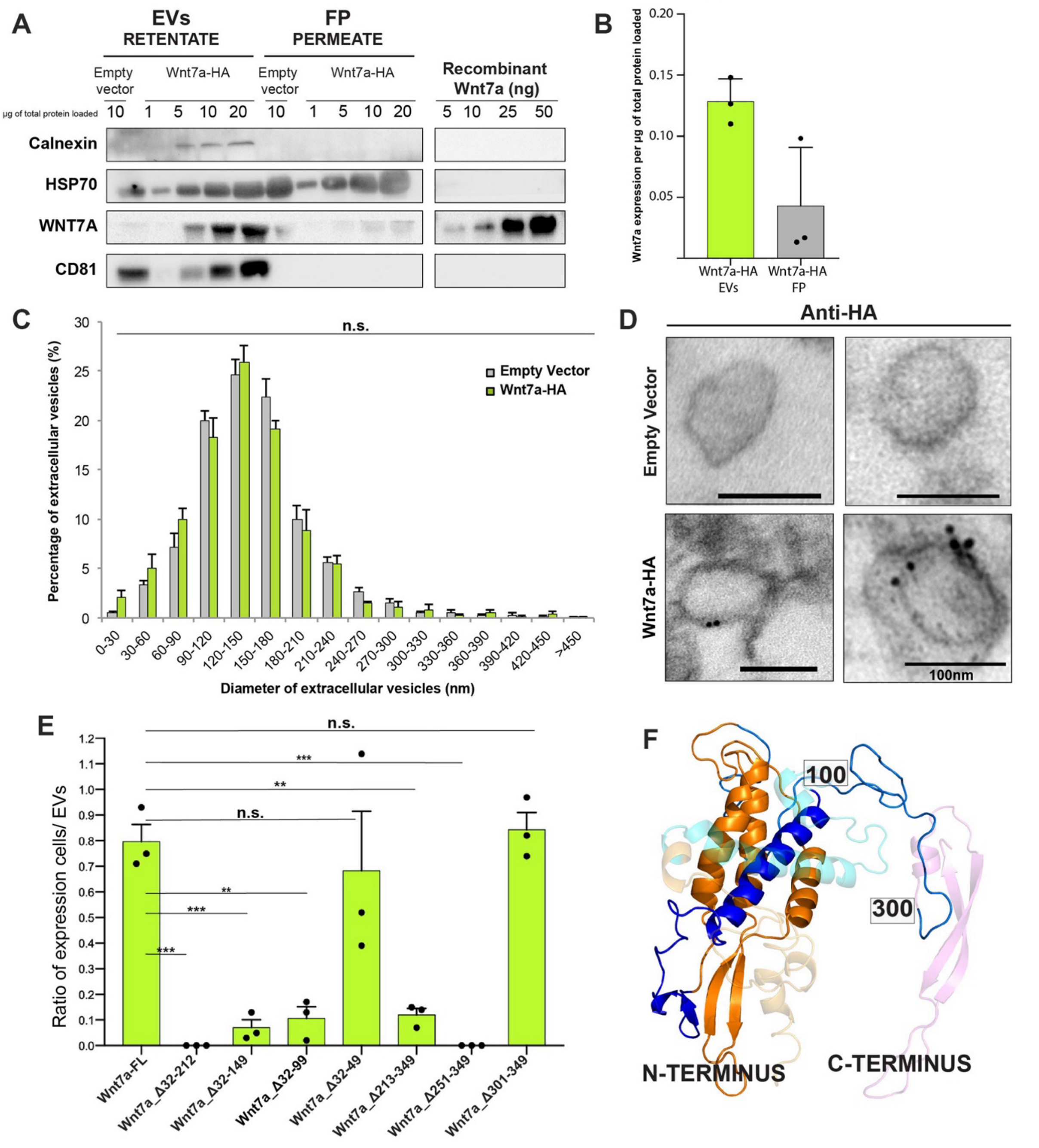

Fig. S2. Wnt7a and deleted mutants are secreted in transfected cells. (**A**) Immunoblot analysis of Wnt7a-EVs derived from HEK293T that are retained inside the TFF cartridge within the retentate fraction, and non-EV Wnt7a passes through the pores of the column and is collected in the permeate fraction. Wnt7a co-purified with EVs together with the exosomal protein CD81. (**B**) Quantification of Wnt7a expression on secreted EVs surface versus non-EV protein secretion derived from HEK293T. (**C**) Relative size distribution analysis of EVs fraction from HEK293T cells comparing EVs derived from Empty Vector transfected

HEK293T cells versus Wnt7a transfected HEK293T cells. (**D**) iTEM of anti-HA labeling of EVs from HEK293T Wnt7a-HA transfected cells, showing HA expression on EVs surface. (**E**) Graph display quantitative secretion analysis of each truncate from Fig. 2b. Data shown as the ratio between EVs and cells fractions. (n=3, Data are mean ± s.e.m., ANOVA test p-value 2.62E-06, TUKEY test **≤0.01, ***≤0.001). (**F**) Wnt7a protein tertiary structure highlighting the Wnt7a minimal structure required for EV secretion. n = 3 biological replicates. Data are mean ± s.e.m.

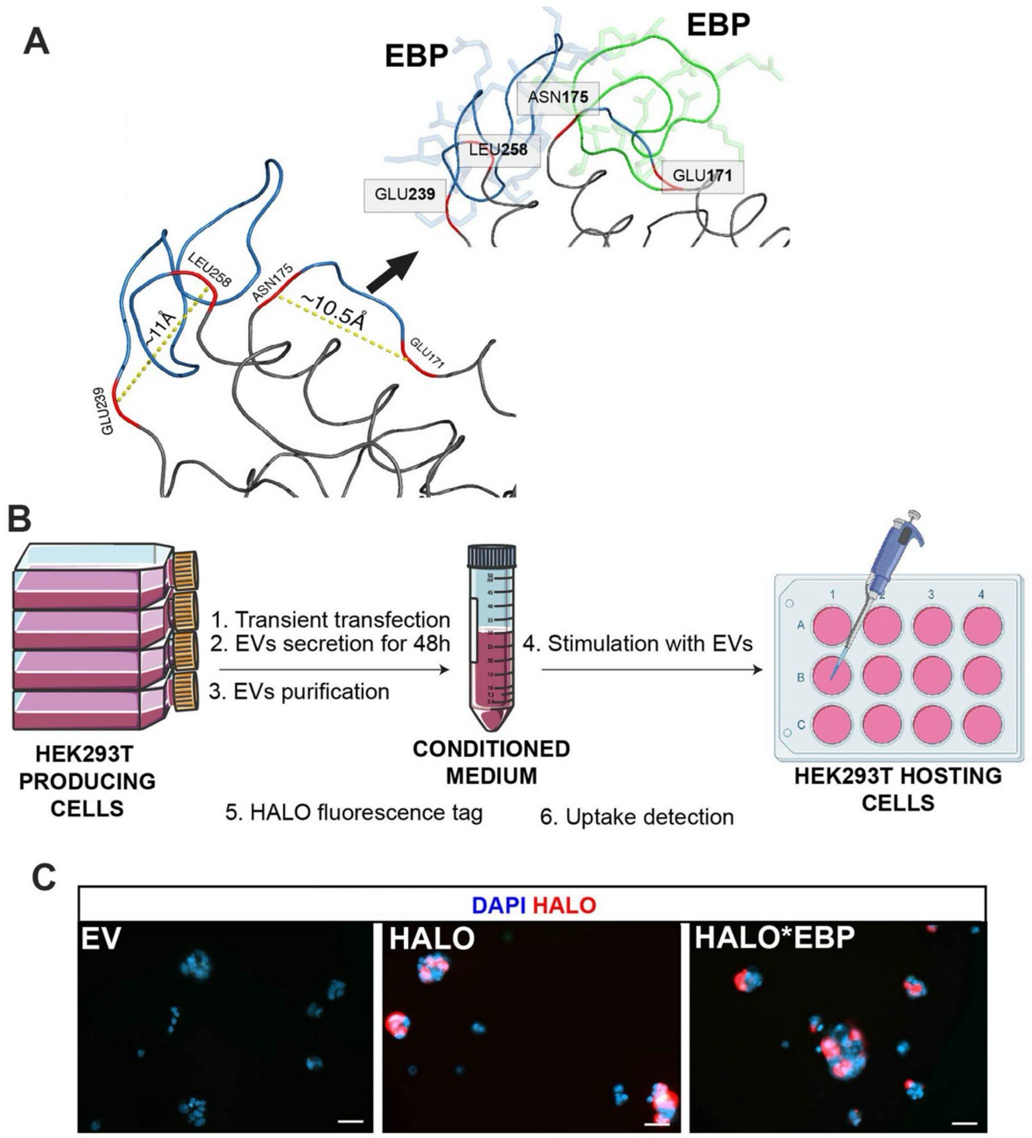

Fig. S3. The EBP is sufficient for secretion of HALO on EVs. (**A**) 3D modeling of EBP insertion in a similar structural space. (A-Left lower) In blue the EBP and the replaced region (AAs 172-174), in red the aminoacids anchoring both unstructured regions. The small difference in Cα-Cα distance of residues anchoring both peptides gives room to swap them, considering as well that are in the same face of the structural surface. (A-Right upper) In green, the EBP modeled into the replaced region (AAs 172-174), side chains in sticks. (**B**) Scheme of the protocol to visualize uptake of HALO*EBP EVs by Image Cytometry. (**C**) Transfected HEK293T cells used to produce EVs, after incubation with HALO fluorescent tag, showing overexpression of HALO and HALO-EBP protein. Scale bar 50μm.

Supplemental Figure S4

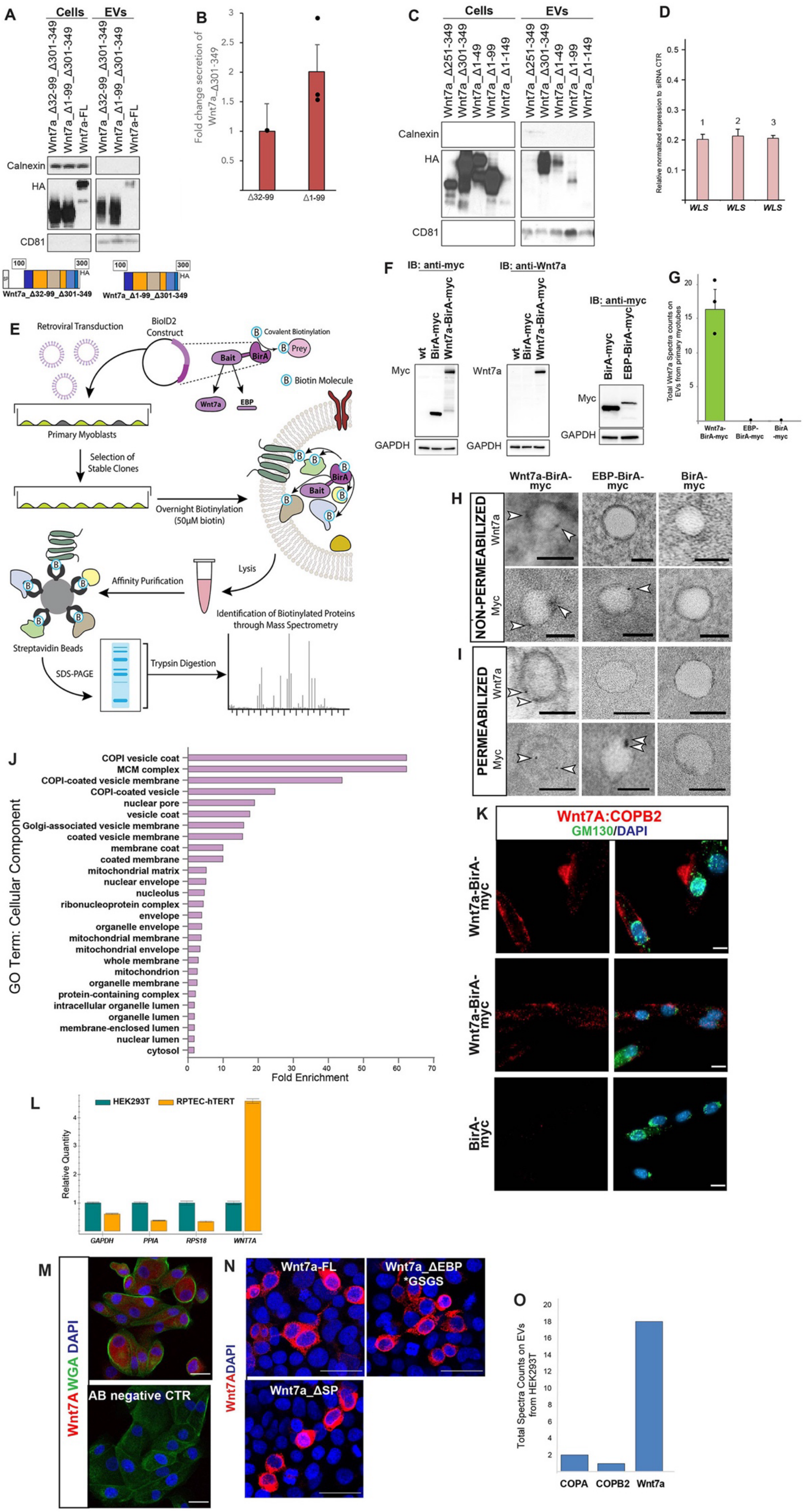

Fig. S4. EBP localization and interactome analysis. (**A**) Immunoblot EVs secretion analysis in HEK293T cells of the minimal Wnt7a structure necessary for EVs secretion from 100-300 aa (right panel) with (Wnt7a_Δ32-99__Δ301-349) and without Signal Peptide (Wnt7a_Δ1-99__Δ301-349). Signal peptide is not required for EVs-Wnt7a secretion. (**B**) Quantification of Wnt7a__Δ301-349 secretion comparing with (Wnt7a_Δ32-99__Δ301-349) and without Signal Peptide (Wnt7a_Δ1-99__Δ301-349) in HEK293T cells. (**C**) Immunoblot secretion analysis in HEK293T cells confirms secretion of the different Wnt7a truncates from Figure 2A without the Signal Peptide. (**D**) qPCR analysis of WLS expression upon siRNA WLS in HEK293T cells. (**E**) Experimental scheme of the BirA analysis protocol. (**F**) Immunoblot analysis of BioID constructs from primary myoblasts expressing WT, BirA-myc tagged, Wnt7a-BirA-myc tagged and EBP-BirA-myc tagged. (**G**) Quantification of Wnt7a mass spectrometry counts for EVs derived from primary myoblasts expressing Wnt7a-BirA-myc tagged, EBP-BirA-myc tagged, and BirA-myc tagged. (**H-I**) iTEM representative images for anti-Wnt7a and anti-Myc labeling of EVs derived from primary myoblasts expressing Wnt7a-BirA-myc tagged, EBP-BirA-myc tagged, and BirA-myc tagged with and without permeabilization. Scale bar 100nm. (**J**) Gene Ontology (GO) term fold enrichment analysis for the gene set displayed in Fig. 4a. The graph displays terms along the hierarchy within the "cellular component" branch, the analysis was performed using ClueGO plugin on Cytoscape software. (**K**) Wnt7a:COPB2 PLA (red) performed in murine primary myotubes either expressing Wnt7a-BirA or BirA. PLA signal was counterstained with GM310 (green), a Golgi Apparatus marker and with DAPI (blue), showing interaction in the plasma membrane area. Scale bar 10 µm. (**L**) qPCR analysis of Wnt7a endogenous expression on RPTEC-hTERT cells. HEK293T cells were used as negative control and GAPDH, Ppia and RPS18 as housekeeping genes. (**M**) Representative immunofluorescence images of Wnt7a endogenous expression on RPTEC-hTERT cells. Counterstaining was done with WGA, a plasma membrane marker and DAPI. No Wnt7a

antibody added was used as a negative control. Scale bar 10μm. (**N**) Representative immunofluorescence images of Wnt7a transfected truncates in HEK293T cells. Scale bar 25μm. (**O**) Quantification of total mass spectrometry counts for Wnt7a-HA, COPA, COPB2 upon HA immunoprecipitation in EVs derived from Wnt7a-HA transfected HEK293T cells. n = 3 biological replicates. Data are mean ± s.e.m.

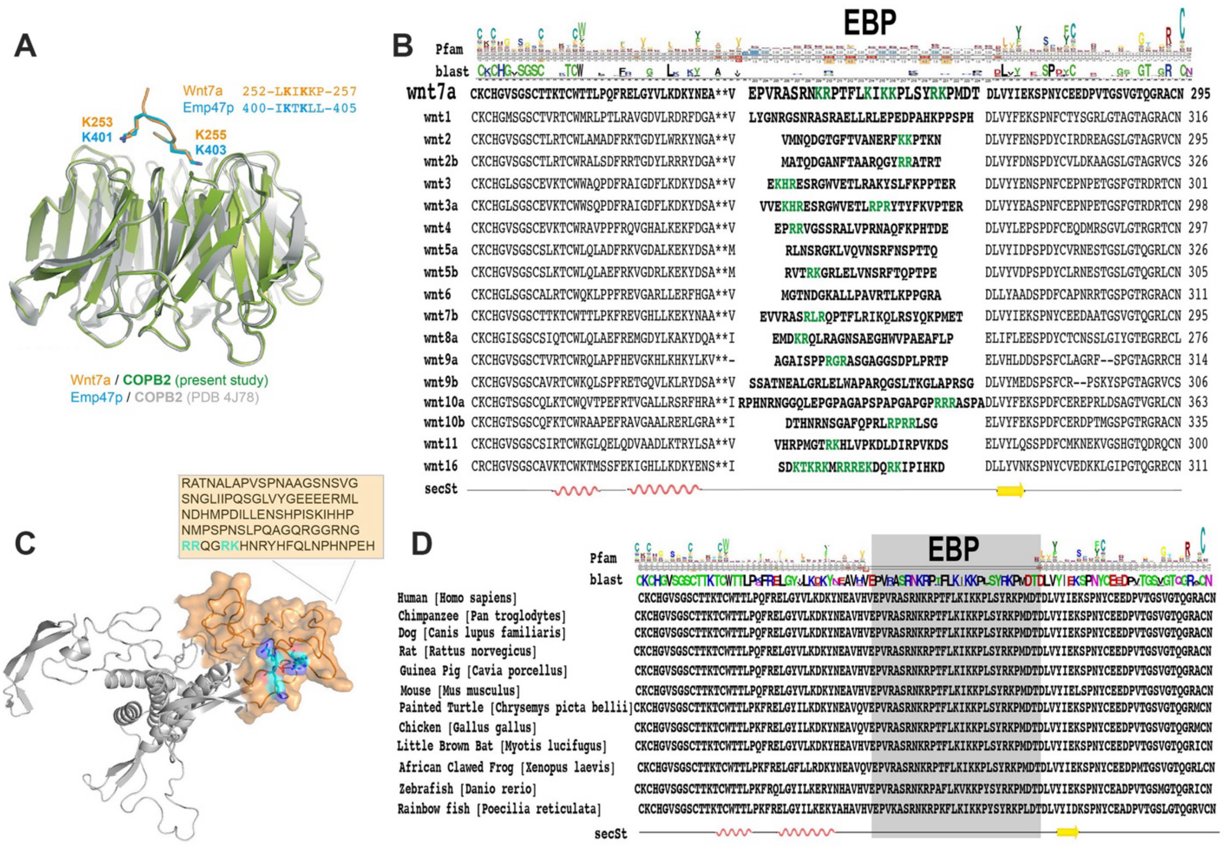

Fig. S5. The EBP is evolutionarily conserved across the Wnt family. (**A**) Overlap of the KTKLL motif of Emp47p (colored blue) and the KTKKP motif of Wnt7a (orange) bound to the COPB2 N-terminal WD-repeat domain. The average root means square deviation (RMSD) for the all-atom pairwise superposition was 0.33 Å. The picture is meant to emphasize that the two KxKxx motifs adopt the same conformation. (**B**) Alignment of Wnt family proteins showing in green the conservation degree of the KKx, RR, KHR, RxR, KR, RR positively charged motifs among the Wnt family. (**C**) Homology model of Wg showing in orange the EBP region and in the left panel the EBP sequence containing the KR and KK motifs in cyan. (**D**) Alignment of EBP-Wnt7a sequences showing the conservation degree across the species.

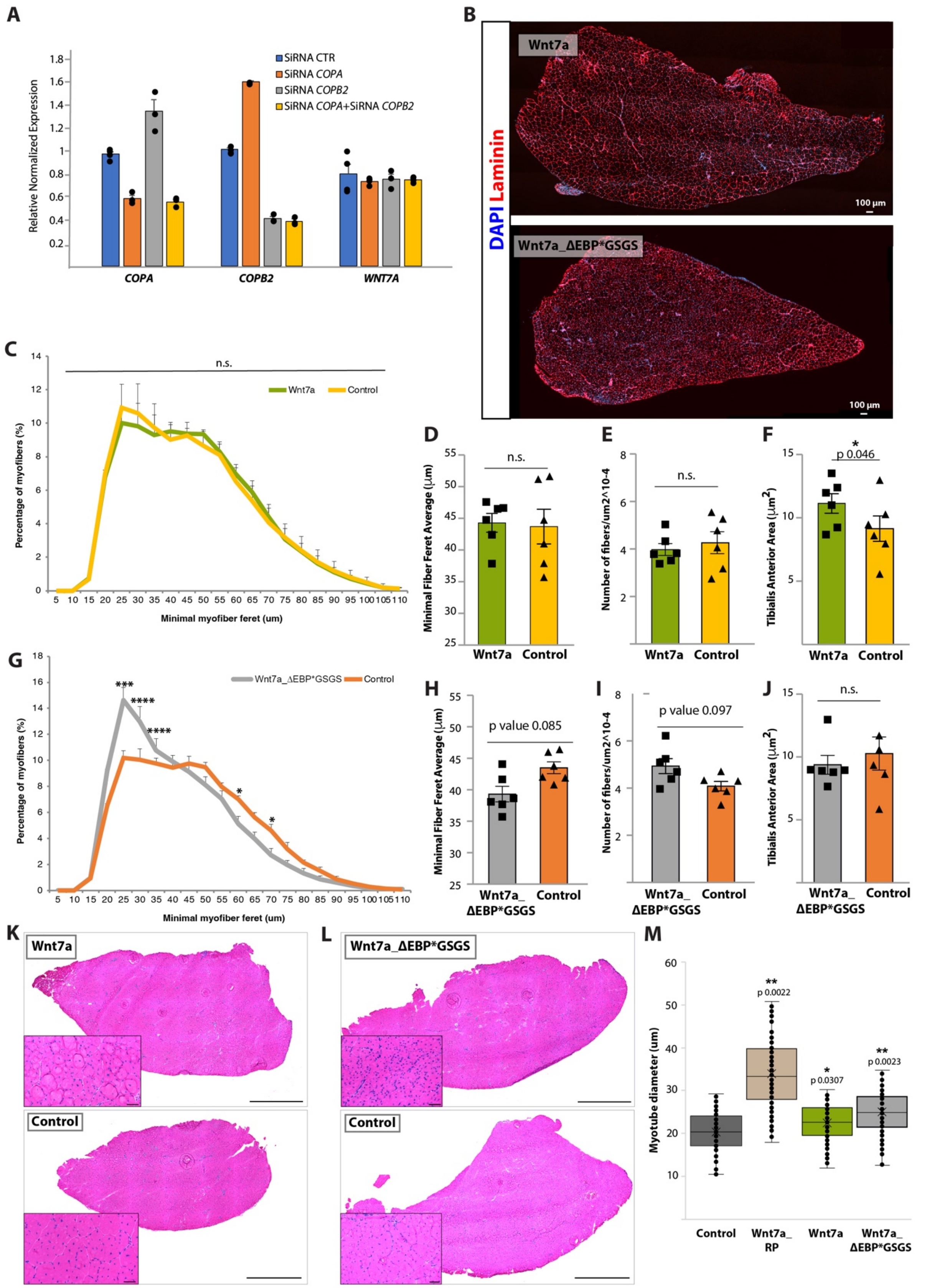

Fig. S6. Coatomer and the EBP domain is required for Wnt7a bioactivity. (**A**) qPCR analysis of COPA, COPB2 and Wnt7a expression upon siRNA COPA, COPB2 in mouse

primary myoblasts. (**B**) Representative immunofluorescence images of the entire TA muscle cryosection 21 days after electroporation of Wnt7a or Wnt7a_ΔEBP*GSGS plasmids. (**C**) Myofiber caliber distribution comparing TA electroporated with Wnt7a (green) vs contralateral control leg (yellow). (**D**) Minimal average feret comparing TA electroporated with Wnt7a (green) vs contralateral control leg (yellow). (**E**) Quantification of fiber number comparing TA electroporated with Wnt7a (green) vs contralateral control leg (yellow). (**F**) Quantification of muscle area comparing TA electroporated with Wnt7a (green) vs contralateral control leg (yellow). (**G**) Myofiber caliber distribution comparing TA electroporated with Wnt7a_ΔEBP*GSGS (grey) vs contralateral control leg (orange). (**H**) Minimal average feret comparing TA electroporated with Wnt7a_ΔEBP*GSGS (grey) vs contralateral control leg (orange). (**I**) Quantification of fiber number comparing TA electroporated with Wnt7a_ΔEBP*GSGS (grey) vs contralateral control leg (orange). (**J**) Quantification of muscle area comparing TA electroporated with Wnt7a_ΔEBP*GSGS (grey) vs contralateral control leg (orange). (**K**) Representative H&E images of the entire TA muscle cryosection 21 days after electroporation, showing and increase in TA muscle area after electroporation of Wnt7a compared to contralateral control leg. Scale bar 1000µm. (**L**) Representative H&E images of the entire TA muscle cryosection 21 days after electroporation, showing an increase in the number of regenerative myofibers and with a smaller caliber size after electroporation of Wnt7a_ΔEBP*GSGS compared to contralateral control leg. Scale bar 1000µm. (**M**) Hypertrophy assay of murine primary myotubes treated with conditioned media from transfected HEK293T cells with the different plasmids Control, Wnt7a, Wnt7a_ΔEBP*GSGS respectively. Wnt7a recombinant protein was used as a positive control. *In vitro* experiments are representative of three independent biological replicates performed in murine primary myoblasts. *In vivo* experiments are representative n=6 mice, mean ± s.e.m. p value determined by two-sided Student's t-test (*p<0.05, **p<0.005).

Table S1. Thermodynamic data from the isothermal titration calorimetry study to characterize the interaction between EBP and COPB2

Injectant	Cell	Kd (μM)	n	ΔH (kcal/mol)	-TΔS (kcal/mol)	ΔG (kcal/mol)
EBP(aa240-257)PVRASRNKRPTFLKIKKP	COPB2(1-304)	11,61	0,97	-13.33±0.71	6.59	-6,74
EBP C-terminal half (aa252-257) LKIKKP	COPB2(1-304)	37,17	0,82	-19.07±2.99	13.02	-6,05
EBP N-terminal half (aa244-250) SRNKRPT	COPB2(1-304)	N.B.				
EBP(K253A) PVRASRNKRPTFLAIKKP	COPB2(1-304)	47,17	1,18	-6.59±1.20	0.69	-5,9
EBP(K255A) PVRASRNKRPTFLKIAKP	COPB2(1-304)	N.B.				
EBP(K256A)PVRASRNKRPTFLKIKAP	COPB2(1-304)	5,99	0,95	-7.63±0.30	0.50	-7,13

Table S2. Data collection and refinement statistics for crystallography.

	COPB2 $_{1\text{-}304}$ / Wnt7a $_{252\text{-}257}$
**Data collection**	
Wavelength [Å]	0.9793
Space group	P1
Resolution [Å]	50.0-1.81 (1.92-1.81)
Cell dimensions	
a, b, c [Å]	1.5, 159.5, 159.6
α, β, γ [º]	107.3, 107.3, 107.4
$CC_{1/2}$ (%)	99.6(31.0)
Completeness (%)	95.5 (91.3)
I/σ	8.53 (0.72)
Number of unique reflexions	1157927(178778)
Redundancy	3.5 (3.6)
**Refinement**	
R-factor (%)	15.9
R-free (%)	17.4
No. atoms:	
Waters	3348
Ions/SO_4	206
Ligand/Glycerol	57
R.m.s deviations:	
Bond lengths (Å)	0.002
Bond angles (º)	1.24
**PDB CODE**	**8R8B**

*Highest resolution shell is shown in parenthesis.

Table S3. Key Resources used in this paper

ANTIBODIES	Source	Identifier
**Calnexin**	Abcam	Cat# ab22595, RRID:AB_2069006
**CD81**	Saint Johns Lab	Cat# STJ96759
**CD9**	SBI	Cat# EXOAB-CD9A-1, RRID:AB_2687469
**COPα**	Santa Cruz Biotechnology	Cat# sc-398099
**COPβ2**	Nobus	Cat# NB600-102, RRID:AB_2081317
**COPβ2**	Cusabio	Cat# PA529993ESR1HU-100UL
**GAPDH**	Sigma-Aldrich	Cat# PLA0302-100UL
**GM130**	Abcam	Cat# ab52649, RRID:AB_880266
**HA**	Bethyl	Cat# A190-108A, RRID:AB_67465
**HALO**	Promega	Cat# GA1110
**HALO**	Promega	Cat# G9211, RRID:AB_2688011
**HSP70**	SBI	Cat# EXOAB-Hsp70A-1, RRID:AB_2687468
**Laminin**	Sigma	Cat# L9393, RRID:AB_477163
**Lamp2**	Abcam	Cat# ab18528, RRID:AB_775981
**Myc**	Bethyl	Cat# A190-105A, RRID:AB_67390
**pMyosin**	DSHB	Cat# MF 20, RRID:AB_2147781
**Wnt7a**	R&D Systems	Cat# AF3008, RRID:AB_2215627
**GM130**	Abcam	Cat# ab52649, RRID:AB_880266
**Goat anti-Mouse IgG (H+L) Secondary Antibody, HRP**	BIO-RAD LABORATORIES (CANADA) LTD	Cat# 170-6516, RRID:AB_11125547
**Goat anti-Rabbit IgG (H+L) Secondary Antibody, HRP**	BIO-RAD LABORATORIES (CANADA) LTD	Cat# 170-6515, RRID:AB_11125142
**Rabbit anti-Goat IgG (H+L) Secondary Antibody, HRP**	Jackson Immunoresearch Labs.	Cat# 305-035-003, RRID:AB_2339400
**0.8nm gold Donkey anti Goat**	Electron Microscopy Sciences	Cat# 25801, RRID:AB_2631210
**12 nm Colloidal Gold AffiniPure Goat Anti-Mouse IgG (H+L) (EM Grade)**	Jackson InmmunoResearch Lab	Cat# 115-205-146, RRID:AB_2338733
**6 nm gold Donkey anti Rabbit**	Jackson InmmunoResearch Lab	Cat# 711-195-152, RRID:AB_2340609
**Goat anti-Rabbit Alexa Fluor 546**	Invitrogen	Cat# A-11010, RRID:AB_2534077
**Goat anti-Rabbit Alexa Fluor 555**	Invitrogen	Cat# A32732, RRID:AB_2633281
**Goat anti-Mouse Alexa Fluor 488**	Invitrogen	Cat# A-11029, RRID:AB_2534088
**Donkey anti-goat Alexa Fluor 488**	Invitrogen	Cat# A-11055, RRID:AB_2534102

CHEMICALS, PEPTIDES, AND RECOMBINANT PROTEIN	Source	Identifier
**Gelatin from cold water fish skin**	Sigma Aldrich	G7765-250ML
**DPBS powder, 1 x 10L**	Wisent	211-410-XK
**Sodium Borohydride**	Sigma-Aldrich	452882-5G

Table S3. Key Resources used in this paper

**Donkey serum**	Abcam	ab7475
**G418**	Fisher	11811-031
**Fetal Bovine Serum**	Wisent	098-150
**Horse Seerum**	Wisent	065-150
**Ham's F-12, 500ml**	Wisent	318-010-CL
**Pen/strep 100X, 100ml**	Wisent	450-201-EL
**Pen/strep 100X, 100ml**	Wisent	450-201-EL
**Puromycin**	Sigma-Aldrich	P8833-100MG
**Wnt7a recombiant protein**	RnD Systems	3008-WN has been discontinued. New one is from PeproTech 120-31-15UG
**DMEM**	Wisent	319-016-CL
**DMEM:F12**	Wisent	319-087-CL
**Triton X-100**	Sigma-Aldrich	T8787-250ML
**Linear polyethylenimine**	Polysciences	23966-1
**8% Paraformaldehyde**	Electron Microscopy Sciences	157-8
**Glycine**	Fisher	BP3815
**BSA**	Sigma-Aldrich	A7906-100G
**DAPI**	Sigma-Aldrich	D8417-5MG
**Hoechst**	Sigma-Aldrich	B2261-100MG
**Permaflour**	Fisher	TA030FM
**Lipofectaamine RNAiMax**	Fisher	13778150
**Takara Ligase Solution**	Takara	6022
**LGK974**	AdooQ	A12816-5
**Magnetic streptavidin beads**	New England Biolabs	S1420S
**4-15% Mini-PROTEAN® TGX Stain-Free™ Protein Gel**	BioRad	4568083S
**Colloidal blue dye**	Thermofisher	LC6025
**VECTASHIELD Antifade Mounting Medium with DAPI**	Vector Laboratories	VECTH1200
**Lipofectamine 2000**	Life Technologies	11668019
**Dynabeads Protein G**	Thermofisher	10004D
**Luria-Bertani broth**	Melford	L24040-5000
**isopropyl-β-D-thiogalactopyranoside**	GoldBio	I2481C100
**phenylmethylsulfonyl fluoride**	Sigma-Aldrich	P7626-5G
**benzamidine**	Sigma-Aldrich	B6506-100G
**Protino Ni-NTA Agarose**	Machenery-Nagel	745400,1
**Imidazol**	Alfa Aesar	A10221.0E

Table S3. Key Resources used in this paper

RECOMBINANT DNA	Source	Identifier
Lenti-III-Ubc-Wnt7a-HA	This paper	N/A
Wnt plasmid kit	Addgene	Cat# 1000000022
pcDNA-hWnt10a-V5	Adgene	Cat# 35939; RRID:Addgene_35939
pcDNA-hWnt16-V5	Adgene	Cat#35942; RRID:Addgene_35942
pcDNA3_HALO*EBP-HA	This paper	N/A
pcDNA3_HALO*EBP	This paper	N/A
pcDNA3_Wnt7a-FL-HA	This paper	N/A
BioID2	This paper	N/A
BioID2-EBP	This paper	N/A
Wnt7a-BioID2	This paper	N/A
pET28-Sumo3 vector	EMBL, Heidelberg	N/A
Lenti-III-Ubc	Applied Biological Materials Inc	G300
pBSM13-Pax7HALO	This paper	N/A
mycBioID2-pBABE-puro	Adgene	Cat#80900, RRID:Addgene_80900
pcDNA3_Wnt7a_Δ32-212	This paper	N/A
pcDNA3_Wnt7a_Δ32-149	This paper	N/A
pcDNA3_Wnt7a_Δ32-99	This paper	N/A
pcDNA3_Wnt7a_Δ32-49	This paper	N/A
pcDNA3_Wnt7a_Δ213-349	This paper	N/A
pcDNA3_Wnt7a_Δ251-349	This paper	N/A
pcDNA3_Wnt7a_Δ301-349	This paper	N/A
pcDNA3_Wnt7a_Δ32-99_Δ301-349	This paper	N/A
pcDNA3_Wnt7a_Δ1-99_Δ301-349	This paper	N/A
pcDNA3_Wnt7a_ΔEBP*GSGS	This paper	N/A
pcDNA3_Wnt7a_Δ1-49	This paper	N/A
pcDNA3_Wnt7a_Δ1-99	This paper	N/A
pcDNA3_Wnt7a_Δ1-149	This paper	N/A
pcDNA3_Wnt7a_Δ3aa*GSG	This paper	N/A
pcDNA3_Wnt7a_Δ3aa*ESP	This paper	N/A
pcDNA3_Wnt7a_Δ213-349*ESP@172	This paper	N/A
pcDNA3_Wnt7a_Δ213-349*ESP	This paper	N/A
pcDNA3_Wnt7a_*ESPΔ213-349	This paper	N/A
pcDNA3_HALO*ESP-HA	This paper	N/A
pcDNA3_HALO*ESP	This paper	N/A
pBABE_Wnt7a-BirA-myc	This paper	N/A
pBABE_Myc-BirA-ESP	This paper	N/A
pcDNA3_Wnt7a_ESP*Scramb	This paper	N/A
pcDNA3_Wnt7a_K247A	This paper	N/A
pcDNA3_Wnt7a_K253A	This paper	N/A
pcDNA3_Wnt7a_K255A	This paper	N/A
pcDNA3_Wnt7a_K256A	This paper	N/A
pcDNA3_Wnt7a_ΔESP*Wnt10a-ESP	This paper	N/A
pcDNA3_Wnt7a_ΔESP*Wnt16-ESP	This paper	N/A
pcDNA3_Wnt10b_ΔESP*GSGS	This paper	N/A
pcDNA3_Wnt10b_RR302AA	This paper	N/A

Table S3. Key Resources used in this paper

OLIGONUCLEOTIDES-RNA	Source	Identifier
**SiRNA-*COPA* mouse**	Horizon Discovery	E-062505-00-0005
**SiRNA-*COPB2* mouse**	Horizon Discovery	E-045228-00-0005
**SiRNA-*COPA* human**	Horizon Discovery	L-011835-00-0005
**SiRNA-*COPB2* human**	Origene	SR306142
**SiRNA-*WLS* human**	Horizon Discovery	LQ-018728-00-0005

CRITICAL COMMERCIAL ASSAYS	Source	Identifier
**HaloTag® Ligands for Super Resolution Microscopy-Janelia 549**	Promega	GA1110
**Mouse on mouse blocking reagent**	Vector Laboratories, Inc	MKB-2213-1
**Silver Enhancement kit**	Electron Microscopy Sciences	25521
**hTERT RPTEC Growth Kit**	ATCC	ACS-4007
**MycoSensor PCR Assay Kit**	Agilent Technologies	302109
**Duolink® In Situ PLA® Probe Anti-Mouse PLUS**	Sigma Aldrich	DUO92001-100RXN
**Duolink PLA probe anti-Mouse MINUS 100**	Sigma Aldrich	DUO92004-100RXN
**Duolink PLA probe anti-Rabbit PLUS 100**	Sigma Aldrich	DUO92002-100RXN
**Duolink® In Situ PLA® Probe Anti-Rabbit MINUS**	Sigma Aldrich	DUO92005-100RXN
**Duolink II Probemaker PLUS**	Sigma Aldrich	DUO92009-1KT
**Duolink II Probemaker MINUS**	Sigma Aldrich	DUO92010-1KT

EXPERIMENTAL MODELS: CELL LINES	Source	Identifier
**HEK293T**	ATCC	Cat#CRL-3216; RRID:CVCL_0063
**RPTEC hTERT1**	ATCC	Cat#crl-4031, RRID:CVCL_K278
**4T1**	ATCC	Cat#CRL-2539, RRID:CVCL_0125

EXPERIMENTAL MODELS: MICE STRAIN	Source	Identifier
**Wnt7a fl/fl**	Originally imported from Dr Thomas Spencer (University of Missouri)	N/A
**Myf5-Cre**	Originally imported from Dr Philipe Soriano now available from The Jackson Laboratory	Strain no. #007893
**C57BL/10ScSN**	The Jackson Laboratory	Strain no. #000476
**C57BL/10ScSn-Dmdmdx/J**	The Jackson Laboratory	Strain no. #001801

Table S3. Key Resources used in this paper

SOFTWARE	Source	Identifier
**Fiji**	NIH	N/A
**Ilustrator 28.1**	Adobe	N/A
**Zen Software**	Zeiss	N/A
**GraphPad Prism**	GraphPad Software	N/A
**FoldX Build model**	FoldX	N/A
**MASCOT 2.6.2**	Matrix Science	N/A
**Scaffold**	Proteome Software	N/A
**SMASH**	The MathWorks, Inc	N/A

DEPOSITED DATA	Source	Identifier
**Raw images for the immunoblots**	This paper	N/A
**Source data of the graphs**	This paper	N/A
**Cristallographic Strucutre with coordinates and structure factors**	Protein Data Bank (http://www.rcsb.org/pdb)	ID code 8R8B

Table S4. Primers used in this study.

Clone	Amino acid sequences	Forward primer	Reverse primer	Tag
**Wnt7a_Δ32-212A3:E17**	MNRKARRCLGHLFLSLGMVYLRIGGFSSVVATCWTTLPQFRELGYVLKDKYNEAVHVE**PVRASRNKRPTFLKIKKP**LSYRKPMDTDLVYIEKSPNYCEEDPVTGSVGTQGRACNKTAPQASGCDLMCCGRGYNTHQYARVWQCNCKFHWCCYVKCNTCSERTEMYTCK**YPYDVPDYA**	gcttctcctcagtggtagctacgtgctggaccacactgccac	aagaattctcaagcgtaatctggaacatcgtatgggtacttgcacgtgtacatctccg	HA
**Wnt7a_Δ32-149**	MNRKARRCLGHLFLSLGMVYLRIGGFSSVVAGGCSADIRYGIGFAKVFVDAREIKQNARTLMNLHNNEAGRKILEENMKLECKCHGVSGSCTTKTCWTTLPQFRELGYVLKDKYNEAVHVE**PVRASRNKRPTFLKIKKP**LSYRKPMDTDLVYIEKSPNYCEEDPVTGSVGTQGRACNKTAPQASGCDLMCCGRGYNTHQYARVWQCNCKFHWCCYVKCNTCSERTEMYTCK**YPYDVPDYA**	aaggatccaccatgaaccggaaagcgcggcgctgcctgggccacctctttctcagcctgggcatggtctacctccggatcggtggcttctcctcagtggtagctggtggctgctctgccgacatc	aagaattctcaagcgtaatctggaacatcgtatgggtacttgcacgtgtacatctccg	HA
**Wnt7a_Δ32-99**	MNRKARRCLGHLFLSLGMVYLRIGGFSSVVAGSREAAFTYAIIAAGVAHAITAACTQGNLSDCGCDKEKQGQYHRDEGWKWGGCSADIRYGIGFAKVFVDAREIKQNARTLMNLHNNEAGRKILEENMKLECKCHGVSGSCTTKTCWTTLPQFRELGYVLKDKYNEAVHVE**PVRASRNKRPTFLKIKKP**LSYRKPMDTDLVYIEKSPNYCEEDPVTGSVGTQGRACNKTAPQASGCDLMCCGRGYNTHQYARVWQCNCKFHWCCYVKCNTCSERTEMYTCK**YPYDVPDYA**	aaggatccaccatgaaccggaaagcgcggcgctgcctgggccacctctttctcagcctgggcatggtctacctccggatcggtggcttctcctcagtggtagctgggagccgggaggctgcgttc	aagaattctcaagcgtaatctggaacatcgtatgggtacttgcacgtgtacatctccg	HA
**Wnt7a_Δ32-49**	MNRKARRCLGHLFLSLGMVYLRIGGFSSVVAAICQSRPDAIIVIGEGSQMGLDECQFQFRNGRWNCSALGERTVFGKELKVGSREAAFTYAIIAAGVAHAITAACTQGNLSDCGCDKEKQGQYHRDEGWKWGGCSADIRYGIGFAKVFVDAREIKQNARTLMNLHNNEAGRKILEENMKLECKCHGVSGSCTTKTCWTTLPQFRELGYVLKDKYNEAVHVE**PVRASRNKRPTFLKIKKP**LSYRKPMDTDLVYIEKSPNYCEEDPVTGSVGTQGRACNKTAPQASGCDLMCCGRGYNTHQYARVWQCNCKFHWCCYVKCNTCSERTEMYTCK**YPYDVPDYA**	aaggatccaccatgaaccggaaagcgcggcgctgcctgggccacctctttctcagcctgggcatggtctacctccggatcggtggcttctcctcagtggtagctgcgatctgccagagccggcccgac	aagaattctcaagcgtaatctggaacatcgtatgggtacttgcacgtgtacatctccg	HA
**Wnt7a_Δ213-349**	MNRKARRCLGHLFLSLGMVYLRIGGFSSVVALGASIICNKIPGLAPRQRAICQSRPDAIIVIGEGSQMGLDECQFQFRNGRWNCSALGERTVFGKELKVGSREAAFTYAIIAAGVAHAITAACTQGNLSDCGCDKEKQGQYHRDEGWKWGGCSADIRYGIGFAKVFVDAREIKQNARTLMNLHNNEAGRKILEENMKLECKCHGVSGSCTTK **YPYDVPDYA**	aaggatccaccatgaaccggaaagcgcggcgctg	aagaattctcaagcgtaatctggaacatcgtatgggtacttggtggtgcacgagcctgac	HA

**Wnt7a_Δ251-349**	MNRKARRCLGHLFLSLGMVYLRIGGFSSVVALGASIICNKIPGLAPRQRAICQSRPDAIIVIGEGSQMGLDECQFQFRNGRWNCSALGERTVFGKELKVGSREAAFTYAIIAAGVAHAITAACTQGNLSDCGCDKEKQGQYHRDEGWKWGGCSADIRYGIGFAKVFVDAREIKQNARTLMNLHNNEAGRKILEENMKLECKCHGVSGSCTTKTCWTTLPQFRELGYVLKDKYNEAVHVE**PVRASRNKRPYPYDVPDYA**	aaggatccaccatgaaccggaaagcgcggcgctg	aagaattctcaagcgtaatctggaacatcgtatgggta ggtgggccgcttgttgcggctg	HA
**Wnt7a_Δ301-349**	MNRKARRCLGHLFLSLGMVYLRIGGFSSVVALGASIICNKIPGLAPRQRAICQSRPDAIIVIGEGSQMGLDECQFQFRNGRWNCSALGERTVFGKELKVGSREAAFTYAIIAAGVAHAITAACTQGNLSDCGCDKEKQGQYHRDEGWKWGGCSADIRYGIGFAKVFVDAREIKQNARTLMNLHNNEAGRKILEENMKLECKCHGVSGSCTTKTCWTTLPQFRELGYVLKDKYNEAVHVE**PVRASRNKRPTFLKIKKP**LSYRKPMDTDLVYIEKSPNYCEEDPVTGSVGTQGRACNKTAPQ **YPYDVPDYA**	aaggatccaccatgaaccggaaagcgcggcgctg	aagaattctcaagcgtaatctggaacatcgtatgggtactggggagccgtcttgttgcag	HA
**Wnt7a_Δ32-99_Δ301-349**	MNRKARRCLGHLFLSLGMVYLRIGGFSSVVAGSREAAFTYAIIAAGVAHAITAACTQGNLSDCGCDKEKQGQYHRDEGWKWGGCSADIRYGIGFAKVFVDAREIKQNARTLMNLHNNEAGRKILEENMKLECKCHGVSGSCTTKTCWTTLPQFRELGYVLKDKYNEAVHVE**PVRASRNKRPTFLKIKKP**LSYRKPMDTDLVYIEKSPNYCEEDPVTGSVGTQGRACNKTAPQ **YPYDVPDYA**	aaggatccaccatgaaccggaaagcgcggcgctgcctgggccacctctttctcagcctgggcatggtctacctccggatcggtggcttctcctcagtggtagctgggagccgggaggctgcgttc	aagaattctcaagcgtaatctggaacatcgtatgggtactggggagccgtcttgttgcag	HA
**Wnt7a_Δ1-99_Δ301-349**	GSREAAFTYAIIAAGVAHAITAACTQGNLSDCGCDKEKQGQYHRDEGWKWGGCSADIRYGIGFAKVFVDAREIKQNARTLMNLHNNEAGRKILEENMKLECKCHGVSGSCTTKTCWTTLPQFRELGYVLKDKYNEAVHVE**PVRASRNKRPTFLKIKKP**LSYRKPMDTDLVYIEKSPNYCEEDPVTGSVGTQGRACNKTAPQ **YPYDVPDYA**	aaggatccaccatggggagccgggaggctgcgttc	aagaattctcaagcgtaatctggaacatcgtatgggtactggggagccgtcttgttgcag	HA
**Wnt7a_ΔEBP*GSGS**	MNRKARRCLGHLFLSLGMVYLRIGGFSSVVALGASIICNKIPGLAPRQRAICQSRPDAIIVIGEGSQMGLDECQFQFRNGRWNCSALGERTVFGKELKVGSREAAFTYAIIAAGVAHAITAACTQGNLSDCGCDKEKQGQYHRDEGWKWGGCSADIRYGIGFAKVFVDAREIKQNARTLMNLHNNEAGRKILEENMKLECKCHGVSGSCTTKTCWTTLPQFRELGYVLKDKYNEAVHVE**GSGS**LSYRKPMDTDLVYIEKSPNYCEEDPVTGSVGTQGRACNKTAPQASGCDLMCCGRGYNTHQYARVWQCNCKFHWCCYVKCNTCSERTEMYTCK **YPYDVPDYA**	vvcaacgaggccgttcacgtggagcctggttcaggttcactgtcgtaccgcaagcccatggacacggac	gtccgtgtccatgggcttgcggtacgacagtgaacctgaaccaggctccacgtgaacggcctcgttg	HA

**Wnt7a_Δ1-49**	AICQSRPDAIIVIGEGSQMGLDECQFQFRNGRWNCSALGERTVFGKELKVGSREAAFTYAIIAAGVAHAITAACTQGNLSDCGCDKEKQGQYHRDEGWKWGGCSADIRYGIGFAKVFVDAREIKQNARTLMNLHNNEAGRKILEENMKLECKCHGVSGSCTTKTCWTTLPQFRELGYVLKDKYNEAVHVE**PVRASRNKRPTFLKIKKP**LSYRKPMDTDLVYIEKSPNYCEEDPVTGSVGTQGRACNKTAPQASGCDLMCCGRGYNTHQYARVWQCNCKFHWCCYVKCNTCSERTEMYTCK **YPYDVPDYA**	aaggatccaccatggcgatctgccagagccggcccgac	aagaattctcaagcgtaatctggaacatcgtatgggtacttgcacgtgtacatctccg	HA
**Wnt7a_Δ1-99**	GSREAAFTYAIIAAGVAHAITAACTQGNLSDCGCDKEKQGQYHRDEGWKWGGCSADIRYGIGFAKVFVDAREIKQNARTLMNLHNNEAGRKILEENMKLECKCHGVSGSCTTKTCWTTLPQFRELGYVLKDKYNEAVHVE**PVRASRNKRPTFLKIKKP**LSYRKPMDTDLVYIEKSPNYCEEDPVTGSVGTQGRACNKTAPQASGCDLMCCGRGYNTHQYARVWQCNCKFHWCCYVKCNTCSERTEMYTCK **YPYDVPDYA**	aaggatccaccatggggagccgggaggctgcgttc	aagaattctcaagcgtaatctggaacatcgtatgggtacttgcacgtgtacatctccg	HA
**Wnt7a_Δ1-149**	GGCSADIRYGIGFAKVFVDAREIKQNARTLMNLHNNEAGRKILEENMKLECKCHGVSGSCTTKTCWTTLPQFRELGYVLKDKYNEAVHVE**PVRASRNKRPTFLKIKKP**LSYRKPMDTDLVYIEKSPNYCEEDPVTGSVGTQGRACNKTAPQASGCDLMCCGRGYNTHQYARVWQCNCKFHWCCYVKCNTCSERTEMYTCK **YPYDVPDYA**	aaggatccaccatgggtggctgctctgccgacatc	aagaattctcaagcgtaatctggaacatcgtatgggtacttgcacgtgtacatctccg	HA
**Wnt7a_Δ3aa*GSG**	MNRKARRCLGHLFLSLGMVYLRIGGFSSVVALGASIICNKIPGLAPRQRAICQSRPDAIIVIGEGSQMGLDECQFQFRNGRWNCSALGERTVFGKELKVGSREAAFTYAIIAAGVAHAITAACTQGNLSDCGCDKEKQGQYHRDEGWKWGGCSADIRYGIGFAKVFVDAREGSGNARTLMNLHNNEAGRKILEENMKLECKCHGVSGSCTTKTCWTTLPQFRELGYVLKDKYNEAVHVE**PVRASRNKRPTFLKIKKP**LSYRKPMDTDLVYIEKSPNYCEEDPVTGSVGTQGRACNKTAPQASGCDLMCCGRGYNTHQYARVWQCNCKFHWCCYVKCNTCSERTEMYTCK**YPYDVPDYA**	caaggtctttgtggatgcccgggagggctcggggaatgcccggactctcatgaacttgcac	gtgcaagttcatgagagtccgggcattccccgagccctcccgggcatccacaaagaccttg	HA
**Wnt7a_Δ3aa*ESP**	MNRKARRCLGHLFLSLGMVYLRIGGFSSVVALGASIICNKIPGLAPRQRAICQSRPDAIIVIGEGSQMGLDECQFQFRNGRWNCSALGERTVFGKELKVGSREAAFTYAIIAAGVAHAITAACTQGNLSDCGCDKEKQGQYHRDEGWKWGGCSADIRYGIGFAKVFVDARE**PVRASRNKRPTFLKIKKP**NARTLMNLHNNEAGRKILEENMKLECKCHGVSGSCTTKTCWTTLPQFRELGYVLKDKYNEAVHVE**PVRASRNKRPTFLKIKKP**LSYRKPMDTDLVYIEKSPNYCEEDPVTGSVGTQGRACNKTAPQASGCDLMCCGRGYNTHQYARVWQCNCKFHWCCYVKCNTCSERTEMYTCK**YPYDVPDYA**	tggcttcttgatcttcaggaaggtgggccgcttgttgcggctggcacgcacaggctcccgggcatccacaaagaccttg	cctgtgcgtgccagccgcaacaagcggcccaccttcctgaagatcaagaagccaaatgcccggactctcatgaacttgc	HA

**Wnt7a_Δ213-349*ESP@172**	MNRKARRCLGHLFLSLGMVYLRIGGFSSVVALGASIICNKIPGLAPRQRAICQSRPDAIIVIGEGSQMGLDECQFQFRNGRWNCSALGERTVFGKELKVGSREAAFTYAIIAAGVAHAITAACTQGNLSDCGCDKEKQGQYHRDEGWKWGGCSADIRYGIGFAKVFVDARE**PVRASRNKRPTFLKIKKP**NARTLMNLHNNEAGRKILEENMKLECKCHGVSGSCTTK **YPYDVPDYA**	aaggatccaccatgaaccggaaagcgcggcgctg	aagaattctcaagcgtaatctggaacatcgtatgggtacttggtggtgcacgagcctgac	HA
**Wnt7a_Δ213-349*ESP**	MNRKARRCLGHLFLSLGMVYLRIGGFSSVVALGASIICNKIPGLAPRQRAICQSRPDAIIVIGEGSQMGLDECQFQFRNGRWNCSALGERTVFGKELKVGSREAAFTYAIIAAGVAHAITAACTQGNLSDCGCDKEKQGQYHRDEGWKWGGCSADIRYGIGFAKVFVDAREIKQNARTLMNLHNNEAGRKILEENMKLECKCHGVSGSCTTK**PVRASRNKRPTFLKIKKP YPYDVPDYA**	aaggatccaccatgaaccggaaagcgcggcgctg	1-gcttgttgcggctggcacgcacaggtcccgatcccttggtggtgcacgagcctgacac 2-agaattctcaagcgta atc tgg aac atc gta tgg gta tggcttcttgatcttcaggaaggtgggccgcttgttgcggctggcacgcac	HA
**Wnt7a_*ESPΔ213-349**	**PVRASRNKRPTFLKIKKP**MNRKARRCLGHLFLSLGMVYLRIGGFSSVVALGASIICNKIPGLAPRQRAICQSRPDAIIVIGEGSQMGLDECQFQFRNGRWNCSALGERTVFGKELKVGSREAAFTYAIIAAGVAHAITAACTQGNLSDCGCDKEKQGQYHRDEGWKWGGCSADIRYGIGFAKVFVDAREIKQNARTLMNLHNNEAGRKILEENMKLECKCHGVSGSCTTK**YPYDVPDYA**	1-cccaccttcctgaagatcaagaagccaggatcgggaatgaaccggaaagcgcggcgctg 2-aggatccccaatgcctgtgcgtgccagccgcaacaagcggcccaccttcctgaagatcaag	aagaattctcaagcgtaatctggaacatcgtatgggtacttggtggtgcacgagcctgac	HA
**HALO*ESP-HA**	MAEIGTGFPFDPHYVEVLGERMHYVDVGPRDGTPVLFLHGNPTSSYVWRNIIPHVAPTHRCIAPDLIGMGKSDKPDLGYFFDDHVRFMDAFIEALGLEEVVLVIHDWGSALGFHWAKRNPERVKGIAFMEFIRPIPTWDEWPEFARETFQAFRTTDVGRKLIIDQNVFIEGTLPMGVVRPLTEVEMDHYREPFLNPVDREPLWRFPNELPIAGEPANIVALVEEYMDWLHQSPVPKLLFWGTPGVLIPPAEAARLAKSLPNCKAVDIGPGLNLLQEDNPDLIGSEIARWLSTLEISGGSGPVRASRNKRPTFLKIKKPYPYDVPDYA	atat aagctt acc atg atataagcttatggaggatctgtactttcag	caagcggcccaccttcctgaagatcaagaagccatac cca tac gat gtt cca gat tac gct tga gaattctt	HA
**HALO*ESP**	MAEIGTGFPFDPHYVEVLGERMHYVDVGPRDGTPVLFLHGNPTSSYVWRNIIPHVAPTHRCIAPDLIGMGKSDKPDLGYFFDDHVRFMDAFIEALGLEEVVLVIHDWGSALGFHWAKRNPERVKGIAFMEFIRPIPTWDEWPEFARETFQAFRTTDVGRKLIIDQNVFIEGTLPMGVVRPLTEVEMDHYREPFLNPVDREPLWRFPNELPIAGEPANIVALVEEYMDWLHQSPVPKLLFWGTPGVLIPPAEAARLAKSLPNCKAVDIGPGLNLLQEDNPDLIGSEIARWLSTLEISGG SGPVRASRNKRPTFLKIKKP	atat aagctt acc atg atataagcttatggaggatctgtactttcag	cttcaggaaggtgggccgcttgttgcggctggcacgcacaggtcccgatccaccggaaatctccagagtag	N/A

**Wnt7a-BirA-myc**	MNRKARRCLGHLFFSLGMVYLRIGGFSSVVALGASIICNKIP GLAPRQRAICQSRPDAIIVIGEGSQMGLDECQFQFRNGRWN CSALGERTVFGKELKVGSREAAFTYAIIAAGVAHAITAACTQ GNLSDCGCDKEKQGQYHRDEGWKWGGCSADIRYGIGFAK VFVDAREIKQNARTLMNLHNNEAGRKILEENMKLECKCHGV SGSCTTKTCWTTLPQFRELGYVLKDKYNEAVHVEPVRASRN KRPTFLKIKKPLSYRKPMDTDLVYIEKSPNYCEEDPVTGSVG TQGRACNKTAPQASGCDLMCCGRGYNTHQYARVWQCNCK FQWCCYVKCNKCSERTEMYTCKTGGSGSGSGSDFKNLIWL KEVDSTQERLKEWNVSYGTALVADRQTKGRGGLGRKWLS QEGGLYFSFLLNPKEFENLLQLPLVLGLSVSEALEEITEIPFS LKWPNDVYFQEKKVSGVLCELSKDKLIVGIGINVNQREIPEE IKDRATTLYEITGKDWDRKEVLLKVLKRISENLKKFKEKSFKE FKGKIESKMLYLGEEVKLLGEGKITGKLVGLSEKGGALILTE EGIKEILSGEFSLRRSLEEQKLISEEDL	**1-** tatagaattcgccaccatgaaccggaa agcgcggcgctgcc **2-** tataaccggtggaagtggaagtggaagt ggaagtgacttcaagaacctgatctggc tg	**1-** tatagtcgaccatatgtatataaccg gtcttgcacgtgtacatctccgtgcg c **2-** tatacatatgtcaaagatcttcctcgg atatgagtttctgctcctcgaggcttct tctcaggctg	MYC
**Myc-BirA-ESP**	MEQKLISEEDLDFKNLIWLKEVDSTQERLKEWNVSYGTALV ADRQTKGRGGLGRKWLSQEGGLYFSFLLNPKEFENLLQLPL VLGLSVSEALEEITEIPFSLKWPNDVYFQEKKVSGVLCELSK DKLIVGIGINVNQREIPEEIKDRATTLYEITGKDWDRKEVLLK VLKRISENLKKFKEKSFKEFKGKIESKMLYLGEEVKLLGEGK ITGKLVGLSEKGGALILTEEGIKEILSGEFSLRRSLEGSGPVR ASRNKRPTFLKIKKP	tatactcgagggatcgggacctgtgcgt gccagccgcaac	gtcgactcatggcttcttgatcttcag gaag	MYC
**Wnt7a_ESP*Scramb**	MNRKARRCLGHLFLSLGMVYLRIGGFSSVVALGASIICNKIP GLAPRQRAICQSRPDAIIVIGEGSQMGLDECQFQFRNGRWN CSALGERTVFGKELKVGSREAAFTYAIIAAGVAHAITAACTQ GNLSDCGCDKEKQGQYHRDEGWKWGGCSADIRYGIGFAK VFVDAREIKQNARTLMNLHNNEAGRKILEENMKLECKCHGV SGSCTTKTCWTTLPQFRELGYVLKDKYNEAVHVE**PNKKLAS PRITFKPKRRV**LSYRKPMDTDLVYIEKSPNYCEEDPVTGSVG TQGRACNKTAPQASGCDLMCCGRGYNTHQYARVWQCNCK FHWCCYVKCNTCSERTEMYTCK**YPYDVPDYA**	tggcgagcccgcgcattacctttaaacc gaaacgccgcgtgctgtcgtaccgcaa gcccatg	ggtttaaaggtaatgcgcgggctcgc cagttttttgttcgGctccacgtgaac ggcctcgttg	HA
**Wnt7a_K247A**	MNRKARRCLGHLFLSLGMVYLRIGGFSSVVALGASIICNKIP GLAPRQRAICQSRPDAIIVIGEGSQMGLDECQFQFRNGRWN CSALGERTVFGKELKVGSREAAFTYAIIAAGVAHAITAACTQ GNLSDCGCDKEKQGQYHRDEGWKWGGCSADIRYGIGFAK VFVDAREIKQNARTLMNLHNNEAGRKILEENMKLECKCHGV SGSCTTKTCWTTLPQFRELGYVLKDKYNEAVHVE**PVRASRN ARPTFLKIKKP**LSYRKPMDTDLVYIEKSPNYCEEPVTGSVGT QGRACNKTAPQASGCDLMCCGRGYNTHQYARVWQCNCKF HWCCYVKCNTCSERTEMYTCK **YPYDVPDYA**	gtgcgtgccagccgcaacgcgcggc ccaccttcctgaagatc	gatcttcaggaaggtgggccgcgcg ttgcggctggcacgcac	HA

**Wnt7a_K253A**	MNRKARRCLGHLFLSLGMVYLRIGGFSSVVALGASIICNKIP GLAPRQRAICQSRPDAIIVIGEGSQMGLDECQFQFRNGRWN CSALGERTVFGKELKVGSREAAFTYAIIAAGVAHAITAACTQ GNLSDCGCDKEKQGQYHRDEGWKWGGCSADIRYGIGFAK VFVDAREIKQNARTLMNLHNNEAGRKILEENMKLECKCHGV SGSCTTKTCWTTLPQFRELGYVLKDKYNEAVHVE**PVRASRN KRPTFLAIKKP**LSYRKPMDTDLVYIEKSPNYCEEDPVTGSVG TQGRACNKTAPQASGCDLMCCGRGYNTHQYARVWQCNCK FHWCCYVKCNTCSERTEMYTCK **YPYDVPDYA**	caagcggcccaccttcctgGCgatca agaagccactgtcgtac	gtacgacagtggcttcttgatcGC caggaaggtgggccgcttg	HA
**Wnt7a_K255A**	MNRKARRCLGHLFLSLGMVYLRIGGFSSVVALGASIICNKIP GLAPRQRAICQSRPDAIIVIGEGSQMGLDECQFQFRNGRWN CSALGERTVFGKELKVGSREAAFTYAIIAAGVAHAITAACTQ GNLSDCGCDKEKQGQYHRDEGWKWGGCSADIRYGIGFAK VFVDAREIKQNARTLMNLHNNEAGRKILEENMKLECKCHGV SGSCTTKTCWTTLPQFRELGYVLKDKYNEAVHVE**PVRASRN KRPTFLKIAKP**LSYRKPMDTDLVYIEKSPNYCEEDPVTGSVG TQGRACNKTAPQASGCDLMCCGRGYNTHQYARVWQCNCK FHWCCYVKCNTCSERTEMYTCK **YPYDVPDYA**	cggcccaccttcctgaagatcGCgaa gccactgtcgtaccgcaag	cttgcggtacgacagtggcttcgcg atcttcaggaaggtgggccg	HA
**Wnt7a_K256A**	MNRKARRCLGHLFLSLGMVYLRIGGFSSVVALGASIICNKIP GLAPRQRAICQSRPDAIIVIGEGSQMGLDECQFQFRNGRWN CSALGERTVFGKELKVGSREAAFTYAIIAAGVAHAITAACTQ GNLSDCGCDKEKQGQYHRDEGWKWGGCSADIRYGIGFAK VFVDAREIKQNARTLMNLHNNEAGRKILEENMKLECKCHGV SGSCTTKTCWTTLPQFRELGYVLKDKYNEAVHVE**PVRASRN KRPTFLKIKAP**LSYRKPMDTDLVYIEKSPNYCEEDPVTGSVG TQGRACNKTAPQASGCDLMCCGRGYNTHQYARVWQCNCK FHWCCYVKCNTCSERTEMYTCK **YPYDVPDYA**	caccttcctgaagatcaagGCgccact gtcgtaccgcaagccc	gggcttgcggtacgacagtggcgc cttgatcttcaggaaggtg	HA
**Wnt7a_ΔESP*Wnt10a-ESP**	MNRKARRCLGHLFLSLGMVYLRIGGFSSVVALGASIICNKIP GLAPRQRAICQSRPDAII VIGEGSQMGLDECQFQFRNGRWNCSALGERTVFGKELKVG SREAAFTYAIIAAGVAHAIT AACTQGNLSDCGCDKEKQGQYHRDEGWKWGGCSADIRYG IGFAKVFVDAREIKQNARTLM NLHNNEAGRKILEENMKLECKCHGVSGSCTTKTCWTTLPQF RELGYVLKDKYNEAVHVEPHNRNGGQLEPGPAGAPSPAPG APGPRRRASDTDLVYIEKSPNYCEEDPVTGSVGTQGRACNK TAPQASGCDLMCCGRGYNTHQYARVWQCNCKFHWCCYVK CNTCSERTEMYTCKYPYDVPDYA	1-aaggatccaccatgaaccggaaagcg cggcgctg<br>2-gct ccg ggc gct ccc ggg ccg cgc cga cgg gcc agc gacacggacctggtgtacatc<br>3-ctg gag ccg ggc cca gcg ggg gca ccc tcg ccg gct ccg ggc gct ccc ggg ccg	1-tgggcccggctccagctggccgcc gttgcggttgtgaggctccacgtgaa cggcctc<br>2-aagaattctcaagcgtaatctggaac atcgtatgggtacttgcacgtgtacat ctccg	HA

**Wnt7a_ΔESP*Wnt16-ESP**	MNRKARRCLGHLFLSLGMVYLRIGGFSSVVALGASIICNKIPGLAPRQRAICQSRPDAIIVIGEGSQMGLDECQFQFRNGRWNCSALGERTVFGKELKVGSREAAFTYAIIAAGVAHAITAACTQGNLSDCGCDKEKQGQYHRDEGWKWGGCSADIRYGIGFAKVFVDAREIKQNARTLMNLHNNEAGRKILEENMKLECKCHGVSGSCTTKTCWTTLPQFRELGYVLKDKYNEAVHVEPKTKRKMRRREKDQRKIPIHDTDLVYIEKSPNYCEEDPVTGSVGTQGRACNKTAPQASGCDLMCCGRGYNTHQYARVWQCNCKFHWCCYVKCNTCSERTEMYTCKYPYDVPDYA	1-aaggatccaccatgaaccggaaagcgcggcgctg2-<br>agg aga gaa aaa gat cag agg aaa ata cca atc cat gacacggacctggtgtacatc	1-atctttttctctcctgcgcattttcctctttgttttaggctccacgtgaacggcctc 2-aagaattctcaagcgtaatctggaacatcgtatgggtacttgcacgtgtacatctccg	HA
**Wnt10b_ΔESP*GSGS**	MLEEPRPRPPPSGLAGLLFLALCSRALSNEILGLKLPGEPPLTANTVCLTLSGLSKRQLGLCLRNPDVTASALQGLHIAVHECQHQLRDQRWNCSALEGGGRLPHHSAILKRGFRESAFSFSMLAAGVMHAVATACSLGKLVSCGCGWKGSGEQDRLRAKLLQLQALSRGKSFPHSLPSPGPGSSPSPGPQDTWEWGGCNHDMDFGEKFSRDFLDSREAPRDIQARMRIHNNRVGRQVVTENLKRKCKCHGTSGSCQFKTCWRAAPEFRAVGAALRERLGAIFI**GSGS**ELVYFEKSPDFCERDPTMGSPGTRGRACNKTSRLLDGCGSLCCGRGHNVLRQTRVERCHCRFHWCCYVLCDECKVTEWVNVCK **YPYDVPDYA**	1-aaggatccaccatgctggaggagccccggcc<br>2-cgg ctg ggc cgg gcc atc ttc att ggttcaggttcagag ctg gtc tac ttt gag aag tct c	1- g aga ctt ctc aaa gta gac cag ctctgaacctgaacc aat gaa gat ggc ccg gcc cag ccg<br>2-aagaattcctaaccggtacgcgtagaatcg	HA
**Wnt10b_RR302AA**	MLEEPRPRPPPSGLAGLLFLALCSRALSNEILGLKLPGEPPLTANTVCLTLSGLSKRQLGLCLRNPDVTASALQGLHIAVHECQHQLRDQRWNCSALEGGGRLPHHSAILKRGFRESAFSFSMLAAGVMHAVATACSLGKLVSCGCGWKGSGEQDRLRAKLLQLQALSRGKSFPHSLPSPGPGSSPSPGPQDTWEWGGCNHDMDFGEKFSRDFLDSREAPRDIQARMRIHNNRVGRQVVTENLKRKCKCHGTSGSCQFKTCWRAAPEFRAVGAALRERLGRAIFIDTHNRNSGAFQPRLRP**AA**LSGELVYFEKSPDFCERDPTMGSPGTRGRACNKTSRLLDGCGSLCCGRGHNVLRQTRVERCHCRFHWCCYVLCDECKVTEWVNVCKYPYDVPDYA	gcc ttc cag ccc cgt ctg cgt ccc gct gcc ctc tca gga gag ctg gtc tac ttt g	c aaa gta gac cag ctc tcc tga gag ggc agc gggacg cag acg ggg ctg gaa ggc	HA
